# Effectiveness and safety of subcutaneous immunotherapy using a depigmented, polymerized extract of cat epithelium in allergic patients: a retrospective, real-world study

**DOI:** 10.3389/falgy.2025.1642315

**Published:** 2025-09-18

**Authors:** María Aránzazu Jiménez-Blanco, María Rosario González-Mendiola, Cosmin Boteanu, Javier Dionicio Elera, Maria Luisa Sánchez-Millán, M. Ruíz-García, Jose Julio Laguna

**Affiliations:** 1 Allergy-Anesthesia Unit, Allergy Department, Hospital Central de la Cruz Roja San José y Santa Adela, Madrid, Spain; 2 Medical Affairs Unit, LETI Pharma, S.L.U., Madrid, Spain; 3 Faculty of Medicine, Universidad Alfonso X El Sabio, Madrid, Spain

**Keywords:** allergen immunotherapy, allergic asthma, allergic rhinitis/rhinoconjunctivitis, cat allergen extract, real-world evidence

## Abstract

**Background:**

Allergy to animal epithelium is on the rise with an estimated 26% of European adults presenting to clinics for suspected inhalant allergy are sensitized to cats, and allergen avoidance measures are often difficult to implement and often ineffective.

**Methods:**

Real-world, retrospective study to evaluate the effectiveness and safety of subcutaneous immunotherapy (SCIT) with depigmented, polymerized cat epithelium extract (Dpg-pol-cat) in adult patients with moderate to severe allergic rhinitis/rhinoconjunctivitis with or without controlled asthma due to cat epithelium sensitization. The primary endpoint was to evaluate the effectiveness of SCIT with Dpg-pol-cat under real-life conditions, as measured by improvement in health-related quality of life (HRQoL) using the validated ESPRINT-15 questionnaire 24 months after treatment initiation.

**Results:**

The study included 28 patients. The median age was 35 years and the median duration of treatment with Dpg-pol-cat was 21.8 months. All patients had a 1-day rush build-up schedule. Significant and sustained improvements in all domains of the ESPRINT-15 questionnaire were observed from month 6 to month 24 of treatment, as well as in reduction of rescue medication use and better asthma control. Specific IgG4 levels increased significantly after 24 months of SCIT, although no significant change was observed in mean anti-Fel d 1 IgG4 levels. Most adverse reactions were local and mild, with systemic reactions, all grade <2 according to the 2010 World Allergy Organization grading system, occurring mainly during the build-up phase.

**Conclusions:**

SCIT with Dpg-pol-cat proved to be effective with an excellent safety profile in this real-world study, making it a good treatment option for patients with rhinitis/rhinoconjunctivitis and asthma due to allergy to cat epithelium.

## Introduction

Allergic diseases, caused by an aberrant immune reaction to certain environmental allergens, have a major negative impact on patients' health-related quality of life (HRQoL) and are the cause of a substantial socioeconomic burden. Due to their dramatic increase in prevalence worldwide, driven in part by increasing environmental risk factors, allergic diseases have become a global public health concern (1–3). It is estimated that allergies and related diseases, ranging from mild to severe conditions, affect up to 30% of the total population, and could reach up to 50% by 2050 (4–7).

The domestic cat, a common household pet worldwide with an average frequency of ownership of 23%, is also a common source of indoor inhalant allergens and the most common risk factor for the development of sensitization and allergic diseases in the general population, including asthma and rhinitis/rhinoconjunctivitis (8–10). Globally, it is estimated that approximately 10%–20% of adults are allergic to cats, a figure that is increasing, and that approximately 20%–30% of patients with respiratory allergies are allergic to cats [(11) and references therein]. In Europe, about 26% of adults seen in the clinic for suspected allergy to inhalant allergens are sensitized to cats (12). A recent article evaluating the sensitization profile of 500 allergic patients from different regions of Germany showed that among all epithelium-sensitized individuals, up to 35.7% (61/171) of patients were sensitized to cat epithelium only, while 46.8% were sensitized to both cat and dog epithelium (80/171) (13). In Spain, allergy to animal epithelium is the third most common cause of respiratory allergy. About 20% of patients diagnosed with allergic rhinoconjunctivitis are sensitized to animal epithelium, with cat being the most common (12.5% of all rhinoconjunctivitis patients and 14.6% of all asthmatic patients). In addition, 10.9% of rhinoconjunctivitis patients and 17% of asthmatics live with cats (14). The glycoprotein Fel d 1 is the major cat allergen involved in the pathogenesis of the disease, interacting with immunoglobulins (Ig) E of over 90% of cat sensitized individuals (15–17).

Preventive measures to avoid exposure to cat epithelium, which are not always feasible, have been shown to be largely ineffective due to the small particle size (up to 23% of total airborne allergen associated with particles <4.7 µm in diameter) and high surface adherence. Several studies on household dust have shown the presence of cat hair particles up to 5 months after the removal of the animal in concentrations enough to induce allergic sensitization (18, 19). On the other hand, up to 34% of cat-allergic patients have never had a cat at home or regular contact with them, showing that cat epithelium is present in high concentrations in public places and workplaces (20).

Allergen-specific immunotherapy (AIT), which involves the progressive administration of increasing doses of allergens, has been shown to be the most effective therapy for aeroallergen respiratory diseases, modifying the underlying disease process and inducing long-term desensitization to the causative allergens (21). Depigmented polymerized allergen extracts have demonstrated their clinical effectiveness while maintaining a good safety profile in several studies conducted with different allergens (22). These modified extracts have reduced allergenicity, i.e., their IgE binding capacity, while maintaining their immunogenicity (23). In addition, large polymerized allergoids dissolve more slowly, thereby creating an antigen depot effect (24). Dpg-pol-cat is a depigmented, polymerized 100% cat epithelium allergen extract for subcutaneous immunotherapy (SCIT). The product is well characterized in terms of allergen content and their relative concentrations, including that of the major cat epithelial allergen Fel d 1, as well as in terms of its mechanism of action (25, 26).

The aim of this retrospective, real-world data study was to generate real-world evidence of the effectiveness and safety of SCIT with Dpg-pol-cat in adult patients with moderate to severe allergic rhinitis/rhinoconjunctivitis with or without controlled asthma due to clinically relevant sensitization to cat epithelium.

## Materials and methods

### Study design and population

This was an observational, retrospective, single-center study to collect data on adult patients with moderate/severe allergic rhinitis/rhinoconjunctivitis with or without controlled asthma due to sensitization to cat epithelium. Participants were recruited during routine visits to the Allergy Department of the Hospital Central de La Cruz Roja in Madrid, Spain. Patients in the effectiveness population were those who had received SCIT for at least 12 months of treatment. Patients in the safety population were those who had received at least one dose of the product. Inclusion criteria included: patients aged ≥18 years with a clinical history consistent with moderate/severe allergic rhinitis/rhinoconjunctivitis according to ARIA (Allergic Rhinitis and its Impact on Asthma) guideline criteria (27) with or without controlled asthma according to GINA (Global Initiative for Asthma) guideline criteria (28) for cat epithelium sensitization, as demonstrated by a positive skin prick test (≥3 mm) to cat epithelium and cat epithelium-specific IgE levels ≥0.35 KU/L; indication for SCIT in the context of routine clinical practice and patients on current or previous SCIT with depigmented, polymerized 100% cat extract for at least 12 months. Exclusion criteria included any contraindication to the use of allergen-specific SCIT in routine clinical practice or according to the product information.

### Endpoints

The primary endpoint was to evaluate the effectiveness of SCIT with Dpg-pol-cat under real-life conditions, as measured by improvement in health-related quality of life (HRQoL) using the validated ESPRINT-15 questionnaire 24 months after treatment initiation. Secondary endpoints included i. to evaluate the effectiveness of SCIT with Dpg-pol-cat by changes in rhinitis HRQoL, symptoms, rescue medication use (as assessed by the daily medication score [dMS], calculated as the sum of oral antihistamines [1 point], topical corticosteroids [2 points] and topical antihistamine/corticosteroid use [3 points]), asthma control and forced expiratory volume (FEV_1_) measurement (both in the asthmatic subgroup) after 6, 12, 18 and/or 24 months of treatment; ii. to evaluate the immunological response to the SCIT after 12 or 24 months of treatment (i.e., measurement of total IgE, cat-specific IgE, anti-Fel d 1 IgE, anti–Fel d 2 IgE, anti-Fel d 4 IgE, total IgG4, cat-specific IgG4, anti-Fel d 1 IgG4, anti-Fel d 2 IgG4 and anti-Fel d 4 IgG4 levels); and (iii) to evaluate the safety of Dpg-pol-cat. The safety assessment included: number of systemic reactions per injection administered [classified according to the 2010 World Allergy Organization (WAO) criteria] and time of occurrence (immediate/late); number of local reactions per injection administered, classified according to size and time of occurrence (immediate/late); number and description of other adverse reactions different from systemic or local.

### Study treatment

Dpg-pol-cat (Depigoid® Cat) is a biologically standardized injectable suspension for subcutaneous administration, composed of depigmented, purified allergen extracts of 100% cat epithelium, polymerized with glutaraldehyde into allergoids, and adsorbed onto aluminum hydroxide. All patients received SCIT in a rush up-dosing schedule (i.e., a single day: two doses of 0.2 and 0.3 ml with a 30-min interval), followed by monthly maintenance injections of 0.5 ml. Treatment was administered for at least 24 months between May 2018 and December 2021. Throughout the study, the patient could have received any concomitant medication according to routine clinical practice or treatment deemed necessary to provide adequate supportive care.

### Analysis of total and specific IgE and IgG

Total IgE and IgG and specific IgE and IgG to cat allergens Fel d 1, Fel d 2 and Fel d 4 were measured using ImmunoCAP (Thermo Fisher Scientific®), following the manufacturer's instructions. The cut-off point for specific IgE was set at ≥0.35 kU/L.

### Statistical analysis

The estimated sample size for the effectiveness analysis (primary endpoint) was estimated at 30 patients and was determined by the total number of patients who attended the Allergology Department of the Hospital between May 2018 and December 2021. Clinical data were collected at baseline and at visits at 6 months, 12 months and 18/24 months (i.e., final visit). The Last Observation Carried Forward (LOCF) imputation method was used to impute missing values. If information from the final visit was missing, the same information from the 12-month visit was imputed for the same patient. Data were recorded in a database specifically designed for the study. Descriptive analyses were carried out for all variables collected. Categorical variables were summarized by frequencies and percentages, whereas continuous variables were summarized using measures of central tendency and dispersion, which may include mean, standard deviation, median, first and third quartiles (Q1; Q3) and extreme values (minimum and maximum). Categorical variables were compared using the Chi-square or Fisher's exact test. For paired data, McNemar's test was used. Numerical variables were compared using the Student's *T*-test, ANOVA or the non-parametric Wilcoxon-Mann–Whitney *U* or Kruskal–Wallis tests. The significance threshold for all bivariate analyses was set at a two-sided *α* = 0.05. All analyses were performed using the statistical software suite SAS version 9.4 (SAS Institute Inc., Cary, NC, USA).

### Ethical considerations

All methods and analyses were performed in compliance with local legal and regulatory requirements, as well as generally accepted research practices described in the latest version of the Declaration of Helsinki and the International Conference of Harmonization-Good Clinical Practice Guidelines. All patients provided written informed consent to participate in the study. All data included in this study were collected as part of routine clinical practice and analyzed retrospectively. In compliance with Organic Law 3/2018, of December 5, on the Protection of Personal Data and the Guarantee of Digital Rights, and Regulation (EU) 2016/679 of the European Parliament and of the Council, of April 27, 2016, all data included in this study were anonymized, a fundamental regulation of patient autonomy and rights and obligations regarding clinical information and documentation, as well as current and applicable regulations.

## Results

### Patient characteristics

Ultimately, data from 28 patients were collected, instead of the 30 initially estimated as the sample size. The median (Q1; Q3) age was 35 (28.3; 39.5) years. There were more women than men (67.9% vs. 32.1%), resulting in a male to female ratio of 0.47. On average, patients had a body mass index (BMI) within the normal range, with a median (Q1; Q3) score of 23.2 (21.1; 25.9). Eight patients (28.6%) were current or former smokers. Most patients with respiratory disease had mild or moderate symptoms, including those with rhinoconjunctivitis (92.9%; 26/28), rhinitis (85.7%; 24/28) and allergic asthma (100.0%; 27/27). All patients with allergic asthma (*n* = 27) had controlled or partially controlled disease, according to clinical criteria. The percentage of patients with atopic dermatitis and food allergy were low, 3.6% and 10.7%, respectively. At baseline, the median (Q1; Q3) levels of cat epithelium-specific IgE were 42.5 (10.5; 78.0) kU/L and total IgE levels were 294.0 (127.0; 726.0) kU/L. All patients had been exposed to cats, and the median (Q1; Q3) exposure time was 36.0 (24.0; 84.0) months. Four patients (14.3%) had been exposed to other animals, in all 4 cases to dogs (Table 1). When analyzing the allergen sensitization profile as determined by skin prick test (SPT), more than half of the population was sensitized to dogs (60.7%) and pollen (75.0%), whereas only a small percentage was allergic to house dust mites (14.3%), molds (7.1%), or pan-allergens (7.1%), including profilin (Pho d 12, 3.6%) and LTP (Pru p 3, 3.6%). The most common pollen allergies identified were to *Lolium perenne* (60.7%), *Phleum pratense* (60.7%), *Olea europea* (53.6%), ash tree (50.0%), and *Cupressus arizonica* (42.9%) (Supplementary Table S1).

**Table 1:** Baseline patient characteristics.

Demographic characteristics	
Age—years^1^	35 (28.3; 39.5)
Women, *n* (%)	19 (67.9%)
Height—m^1^	1.7 (1.6; 1.7)
Weight—kg^1^	62.5 (57.0; 76.5)
BMI—kg/m^2^^1^	23.2 (21.1; 25.9)
Current or former smoker, *n* (%)	8 (28.6)
Clinical and laboratory parameters	
Rhinoconjunctivitis, *n* (%)	
Total	28 (100.0)
Mild	5 (17.9)
Moderate	21 (75.0)
Severe	2 (7.1)
Rhinitis, *n* (%)	
Total	28 (100.0)
Mild	5 (17.9)
Moderate	19 (67.9)
Severe	4 (14.3)
Allergic asthma, *n* (%)	
Total	27 (100.0)
Mild	7 (25.9)
Moderate	20 (74.1)
Severe	0 (0.0)
Asthma control, *n* (%)^2^	
Total	27 (100.0)
Controlled	8 (29.6)
Partially controlled	19 (70.4)
Uncontrolled	0 (0.0)
Atopic dermatitis, *n* (%)	
Total	28 (100.0)
Yes	1 (3.6)
Food allergy, *n* (%)	
Total	28 (100.0)
Yes	3 (10.7)
Cat-specific IgE—kU/L	
*N*	27
Median (Q1; Q3)	42.5 (10.5; 78.0)
Missing, *n*	1
Total IgE—kU/L	
*N*	27
Median (Q1; Q3)	294.0 (127.0; 726.0)
Missing, *n*	1
SCIT treatment duration—months	
Median (Q1; Q3)	21.8 (18.1; 25.2)
Missing, *n*	2

Animal exposure	
Actual cat exposure, *n* (%)	
Total	28 (100.0)
Yes	28 (100.0)
Animal sex, *n* (%)^3^	
Total	17 (100.0)
Female	8 (47.1)
Missing	11
Onset of cat exposure—months	
*N*	27
Median (Q1; Q3)	36 (24.0; 84.0)
Min–Max	12-120
Missing, *n*	1
Exposure to other animals, *n* (%)	
Total	28 (100.0)
Yes	4 (14.3)
Other animals specified, *n* (%)	
Total	4 (100.0)
Dog	4 (100.0)

1 Results expressed as median (Q1; Q3).

2 Asthma control according to clinical criteria.

3 The same record may have more than one animal of different sexes, so the percentages may not add up to 100%.

### Treatment-related improvement in rhinitis HRQoL

All patients had a rapid, 1-day rush build up schedule. The median (Q1; Q3) time of SCIT with Dpg-pol-cat was 21.8 (18.1; 25.2) months. SCIT treatment resulted in a sustained, statistically significant improvement in allergic rhinitis HRQoL (i.e., patients' perception of rhinitis symptoms) as measured by the ESPRINT-15 questionnaire, from 6 months to the final visit. The median (Q1; Q3) questionnaire score decreased significantly from 36.0 (18.0; 52.0) points at baseline, to 35.0 (10.0; 45.0) at 6 months (*p* = 0.0052) and to 13.0 (8.0; 35.0) at 12 months (*p* = 0.0002), with a slight recovery to 16.0 (7.0; 31.0) points at the final visit (baseline vs. final visit, *p* = 0.0009; 12 months vs. final visit, *p* = 0.0470). Overall, the median questionnaire score decreased by 20 points from baseline to the final visit (*p* = 0.0004). When the patients were asked to rate their overall HRQoL, considering only their rhinitis, the proportion of patients who reported feeling better increased progressively during SCIT treatment. The percentage of patients feeling good, very good or excellent increased from 76.8% at baseline to 81.8% at 6 months (*p* = 0.0009), to 89.5% at 12 months (*p* = 0.0006), and to 90.9% at the final visit (*p* = 0.2211). Overall, the proportion of patients feeling better after SCIT treatment increased by approximately 13% from baseline to the LOCF (78.6% to 88.9%, *p* = 0.0082) (Table 2).

**Table 2:** Health-related quality of life (ESPRINT-15 questionnaire).

Variable	Visit								
	Baseline	6 months	*p*-value^1^	12 months	*p*-value	18/24 months^2^	*p*-value	LOCF^3^	*p*-value
ESPRINT-15 questionnaire									
*N*	28	10	0.0052	17	0.0002	22	0.0009	26	0.0004
Mean (SD)	35.39 (21.09)	31.20 (22.08)		21.00 (17.49)		18.95 (15.64)		19.08 (15.53)	
Median (Q1; Q3)	36.0 (18.0; 52.0)	35.0 (10.0; 45.0)		13.0 (8.0; 35.0)		16.0 (7.0; 31.0)		16.0 (7.0; 31.0)	
Min–Max	3.0–82.0	2.0–63.0		1.0–48.0		0.0–54.0		0.0–54.0	
Missing, *n*	0	18		11		6		2	
Final question^4^									
Total, *n* (%)	28 (100.0)	11 (100.0)	0.0053	19 (100.0)	0.0004	22 (100.0)	0.0685	27 (100.0)	0.0027
Very bad, *n* (%)	0 (0.0)	0 (0.0)		0 (0.0)		0 (0.0)		0 (0.0)	
Bad, *n* (%)	1 (3.6)								
Fair, *n* (%)	5 (17.9)	2 (18.2)		2 (10.5)		2 (9.1)		3 (11.1)	
Good, *n* (%)	17 (60.7)	6 (54.5)		10 (52.6)		15 (68.2)		16 (59.3)	
Very Good, *n* (%)	3 (10.7)	2 (18.2)		5 (26.3)		3 (13.6)		5 (18.5)	
Excellent, *n* (%)	2 (7.1)	1 (9.1)		2 (10.5)		2 (9.1)		3 (11.1)	
Missing, *n*	0	17		9		6		1	
Final question (two categories)^2^									
Total, *n* (%)	28 (100.0)	11 (100.0)	0.0009	19 (100.0)	0.0006	22 (100.0)	0.2211	27 (100.0)	0.0228
Very bad/Bad/Fair, *n* (%)	6 (21.4)	2 (18.2)		2 (10.5)		2 (9.1)		3 (11.1)	
Good/Very good/Excellent, *n* (%)	22 (78.6)	9 (81.8)		17 (89.5)		20 (90.9)		24 (88.9)	
Missing, *n*	0	17		9		6		1	

LOCF, last observation carried forward.

1 Student *t*-test for paired data.

2 Final visit.

3 If information from the last visit was missing, the same information from the 12-month visit was assigned to the same patient.

4 Final question was: In general, considering only your rhinitis and no other illness, how would you rate your health?

### Severity of clinical symptoms

**Figure 1:**
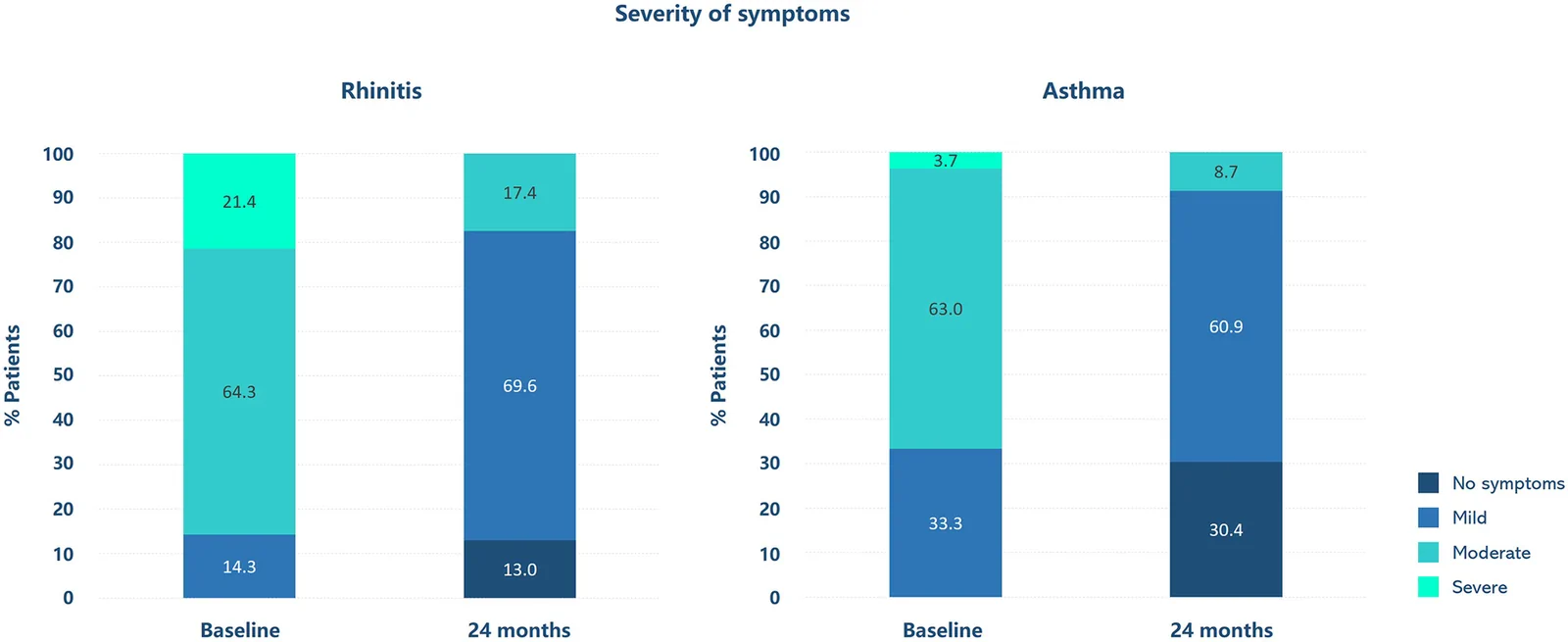
Percentage of patients with different severity of rhinitis (left panel) and asthma (right panel) symptoms at baseline and after 24 months of allergen-specific immunotherapy. Symptom severity is color-coded as shown in the box at the bottom right of the figure.

**Figure 2:**
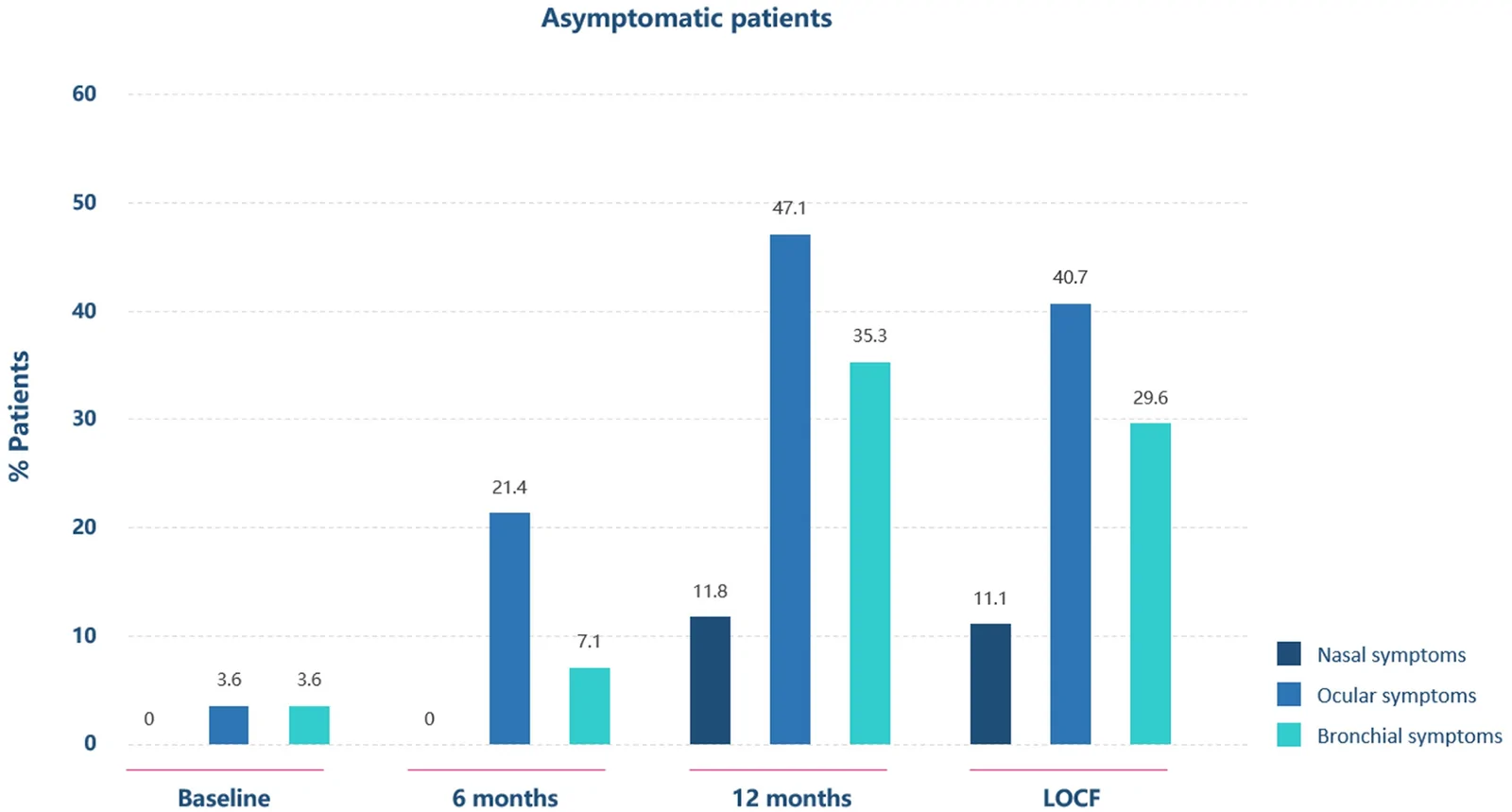
Evolution of the percentage of patients without nasal, ocular and bronchial symptoms at baseline and after 6 months, 12 months and LOCF (Last Observation Carried Forward) of allergen-specific immunotherapy. Percentages are shown above each histogram bar. Symptoms are color-coded as shown in the box at the bottom right of the figure.

At baseline, most patients had moderate or severe symptoms of rhinitis (85.7%), rhinorrhea (82.2%), nasal itchiness (71.4%), nasal obstruction (71.4%), dyspnea (70.3%), asthma (66.7%), and ocular itchiness (53.6%). After SCIT treatment with Dpg-pol-cat, although the changes from baseline were not statistically significant except for wheezing (*p* = 0.041), most patients were asymptomatic or had mild symptoms at the LOCF, including asthma (92.3%), rhinitis (81.5%), conjunctivitis (66.3%), dyspnea (96.3%), and wheezing (100.0%) (Figure 1 and Supplementary Table S2). Comparing the presence of clustered symptoms at baseline (including mild, moderate, and severe symptoms) with that at the LOCF, the percentage of asymptomatic patients increased from none to 11.1% (*n* = 3) for nasal symptoms (including rhinorrhea, nasal itching and nasal obstruction), from 3.6% to 40.7% (*n* = 1 vs. 11) for ocular symptoms (including eye itching, tearing and eye redness), and from 3.6% to 29.6% (*n* = 1 vs. 8) for bronchial symptoms (including cough, dyspnea and wheezing) (Figure 2). For symptom improvement, defined as a decrease in symptom severity rating, regardless of step, data were available for 27 patients at the end of the study (1 patient lost to follow-up). Comparing baseline and LOCF data, most patients experienced improvement in all symptoms, with statistically significant changes in tearing (63%; *p* = 0.031), eye redness (48.1%; *p* = 0.001) and wheezes (70.4%; *p* = 0.006) (Supplementary Table S3).

### Use of rescue medication

All patients used medication for rhinitis prior to starting SCIT with Dpg-pol-cat to manage symptoms. The mean dMS, which was estimated at 1.79 (SD 0.50) before SCIT, significantly decreased after treatment to 0.96 (SD 0.79) at the LOCF (*p* < 0.0001). Regarding the use of specific medications, antihistamines were reduced by 31.0% (*p* = 0.1610), antileukotrienes by 14.3% (*p* = 0.0015), short-acting B2 agonists (SABAs) by 3.6% (*p* < 0.0001), nasal steroids by 36% (*p* = 0.0202), and inhaled steroids plus long-acting B2 agonists (LABAs) by 28.6% (*p* = 0.0052) (Table 3 and Supplementary Table S4).

**Table 3:** Daily medication score, asthma control and lung function during follow-up.

Variable	Visit								
	Baseline	6 months	*p*-value^1^	12 months	*p*-value	18/24 months^2^	*p*-value	Final LOCF^3^	*p*-value
Daily medication score									
dMS^4^^,^^5^									
*N* (%)	28 (100.0)	28 (100.0)	0.0057	28 (100.0)	0.0004	28 (100.0)	<0.0001	28 (100.0)	<0.0001
Mean (SD)	1.79 (0.50)	1.54 (0.69)		1.21 (0.83)		0.96 (0.79)		0.96 (0.79)	
Median (Q1; Q3)	2.0 (2.0; 2.0)	2.0 (1.0; 2.0)		1.0 (0.5; 2.0)		1.0 (0.0; 2.0)		1.0 (0.0; 2.0)	
Min–Max	0.0-2-0	0.0-2-0		0.0-2-0		0.0-2-0		0.0-2-0	
Missing, *n*	0	0		0		0		0	
Asthma control									
ACT questionnaire									
*N* (%)	27 (100.0)	17 (100.0)	0.0051	21 (100.0)	0.0201	18 (100.0)	0.0028	26 (100.0)	0.0121
Mean (SD)	19.07 (4.68)	21.63 (2.87)		21.95 (3.40)		22.44 (2.50)		22.00 (3.20)	
Median (Q1; Q3)	20 (17.0; 22.0)	22 (19.5; 24.0)		23.0 (21.0; 24.0)		22.5 (22.0; 24.0)		22.5 (21.0; 24.0)	
Min–Max	6.0-25.0	16.0-25.0		11.0-25.0		16.0-25.0		11.0-25.0	
Missing, *n*	0	11		6		9		1	
Asthma control^6^									
*N* (%)	27 (100.0)	16 (100.0)	0.3082	20 (100.0)	0.6026	18 (100.0)	0.2263	25 (100.0)	0.4826
Good, *n* (%)	11 (40.7)	11 (68.8)		16 (80.0)		15 (83.3)		20 (80.0)	
Partial, *n* (%)	7 (25.9)	2 (12.5)		2 (10.0)		1 (5.6)		2 (8.0)	
Bad, *n* (%)	9 (33.3)	3 (18.8)		2 (10.0)		2 (11.1)		3 (12.0)	
Missing, *n*	0	11		7		9		2	
Lung function									
FEV_1_ (Liters)									
*N* (%)	26 (100.0)	5 (100.0)	0.0779	12 (100.0)	0.095	12 (100.0)	0.8532	15 (100.0)	0.6711
Mean (SD)	3.47 (0.57)	3.39 (0.38)		3.48 (0.68)		3.55 (0.34)		3.57 (0.61)	
Median (Q1; Q3)	3.3 (3.1; 3.8)	3.3 (3.1; 3.4)		3.3 (3.1; 3.8)		3.5 (3.3; 3.8)		3.5 (3.3; 3.8)	
Min–Max	2.6–5.3	3.1–4.0		2.3–5.1		3.2–4.3		2.3–5.1	
Missing, *n*	1	22		15		15		12	
FEV_1_ (%)									
*N* (%)	26 (100.0)	5 (100.0)	0.0416	11 (100.0)	0.2719	12 (100.0)	0.4338	16 (100.0)	0.8215
Mean (SD)	95.19 (7.29)	88.40 (6.07)		92.64 (8.46)		96.92 (4.91)		95.13 (8.08)	
Median (Q1; Q3)	98 (93.0; 100.0)	89 (87.0; 92.0)		93 (89.0; 99.0)		99 (96.5; 100.0)		98 (93.0; 100.0)	
Min–Max	71.0–100.0	79.0–95.0		71.0–100.0		85.0–100.0		71.0–100.0	
Missing, *n*	1	22		16		15		12	

dMS, daily medication score; FEV_1_, forced expiratory volume in one second; LOCF, last observation carried forward.

1 Student *t*-test for paired data or Chi-square according to type of data.

2 Final visit.

3 If information from the last visit was missing, the same information from the 12-month visit was assigned to the same patient.

4 The daily medication score (dMS) was calculated as the sum of oral antihistamines, topical corticosteroids, and topical antihistamine/corticosteroids used.

5 dMS punctuation = 1 point for subjects with oral antihistamines; 2 points for subjects with topical corticosteroids; 3 points for subjects with topical corticosteroids and topical antihistamines.

6 Good control (ACT ≥ 20), partial control (15 < ACT <20), bad control (ACT ≤ 15).

### Asthma control

Asthma control was assessed in asthmatic patients using the validated Asthma Control Test (ACT). The ACT mean score, which was estimated to be 19.07 (SD: 4.68) at baseline, progressively and significantly improved during the SCIT treatment with Dpg-pol-cat, reaching mean scores of 21.63 (SD: 2.87) at 6 months (*p* = 0.0051), 21.95 (SD 3.40) at 12 months (*p* = 0.0201) and 22.00 (SD: 3.20) at the LOCF (*p* = 0.0121). Overall, the percentage of patients with good asthma control (i.e., ACT score ≥20) increased from 40.7% at baseline to 80.0% at the LOCF, whereas the percentage of patients with partial (i.e., 15 < ACT score < 20) and bad (i.e., ACT score ≤15) control decreased from 25.9% to 8.0% and from 33.3% to 12.0%, respectively (Table 3).

### Lung function

Compared to baseline, a slight increase in FEV_1_ was observed at 12 months, final visit and LOCF, although values were close to initial values [92.6% (SD: 8.46), 96.9% (SD: 4.91) and 95.1% (SD: 8.08), respectively]. Nevertheless, a decrease was observed at 6 months, probably due to the high number of missing data, as patients were treated during the COVID-19 pandemic and spirometry data were not available (Table 3).

### Immunological data

IgE levels were measured at baseline and at each follow-up visit. There were no significant changes during the study period in total IgE, cat epithelium-specific IgE, anti-Fel d 1, anti-Fel d 2, or anti-Fel d 4 IgE antibodies. No significant associations were found between specific IgE levels (Fel d 1, Fel d 2, Fel d 4; mono- or oligosensitized) and the severity of asthma, rhinitis, or atopic dermatitis, nor with the classification of asthma or rhinitis (data for Fel d 2 and Fel d 4 not shown). Cat epithelium-specific IgG4 antibodies increased significantly from baseline to the final visit, from 3.84 to 4.43 kU/L (*p* = 0.0092). No significant changes were observed in total IgG4, anti-Fel d 1, anti-Fel d 2 or anti-Fel d 4 IgG4 antibodies (Supplementary Table S5).

### Safety

Thirteen out of the 28 patients (46.4%) had a total of 20 adverse reactions after initiation of SCIT with Dpg-pol-cat, of which 16 were local reactions (80.0%) and 2 were systemic reactions (15.5%). One patient (5.0%) had both local and systemic reactions. All local reactions had a late onset, with 10 (62.5%) occurring during the up-dosing and 6 (37.5%) during the maintenance phase. Most local reactions (*n* = 15; 93.8%) were mild (*n* = 7; 43.8%) or moderate (*n* = 8; 50.0%), with only one reaction (6.2%) classified as severe. Three additional systemic reactions occurred during the maintenance phase, for a total of five systemic reactions. In most cases, no treatment adjustment was required during follow-up. Only 1 patient required a dose reduction and 7 patients received premedication with antihistamines. According to the 2010 WAO criteria, there were 2 grade I cases (40.0%) and 3 grade II cases (60.0%), and no grade III-IV reactions. All patients recovered from systemic reactions and only one patient discontinued the treatment (Table 4).

**Table 4:** Adverse events.

Variable	Baseline	6 months	12 months	18/24 months^1^
Subjects with AR, *n* (%)				
Total	28 (100.0)	25 (100.0)	24 (100.0)	23 (100.0)
Yes	13 (46.4)	3 (12.0)	2 (8.3)	0 (0.0)
No	15 (53.6)	22 (88.0)	22 (91.7)	23 (100.0)
Missing	0	3	4	5
AR, *n* (%)	20 (100.0)	8 (100.0)	2 (100.0)	0 (0.0)
Phase of SCIT, *n* (%)				
Up-dosing	13 (65.0)	0 (0.0)	0 (0.0)	0 (0.0)
Maintenance	7 (35.0)	8 (100.0)	2 (100.0)	0 (0.0)
Type of AR, *n* (%)				
Local	16 (80.0)	7 (87.5)	1 (50.0)	0 (0.0)
Local and systemic^2^	1 (5.0)	0 (0.0)	0 (0.0)	0 (0.0)
Systemic	3 (15.0)	1 (12.5)	1 (50.0)	0 (0.0)
Local AR, *n* (%)	16 (100.0)	7 (100.0)	1 (100.0)	0 (0.0)
Phase of SCIT, *n* (%)				
Up-dosing	10 (62.5)	0 (0.0)	0 (0.0)	0 (0.0)
Maintenance	6 (37.5)	7 (100.0)	1 (100.0)	0 (0.0)
Time of onset				
Immediate	0 (0.0)	0 (0.0)	0 (0.0)	0 (0.0)
Late	16 (100.0)	7 (100.0)	1 (100.0)	0 (0.0)
Seriousness				
Mild	7 (43.8)	5 (71.4)	1 (100.0)	0 (0.0)
Moderate	8 (50.0)	2 (28.6)	0 (0.0)	0 (0.0)
Systemic AR, *n* (%)	3 (100.0)	1 (100.0)	1 (100.0)	0 (0.0)
Phase of SCIT				
Up-dosing	2 (66.7)	0 (0.0)	0 (0.0)	0 (0.0)
Maintenance	1 (33.3)	1 (100.0)	1 (100.0)	0 (0.0)
Time of onset				
Immediate	0 (0.0)	1 (100.0)	0 (0.0)	0 (0.0)
Late	3 (100.0)	0 (0.0)	1 (100.0)	0 (0.0)
Seriousness (WAO 2010), *n* (%)				
Grade 1	1 (33.3)	0 (0.0)	1 (100.0)	0 (0.0)
Grade 2	2 (67.7)	1 (100.0)	0 (0.0)	0 (0.0)
Grade 3	0 (0.0)	0 (0.0)	0 (0.0)	0 (0.0)
Grade 4	0 (0.0)	0 (0.0)	0 (0.0)	0 (0.0)
Grade 5	0 (0.0)	0 (0.0)	0 (0.0)	0 (0.0)

AR, adverse reaction; SCIT, subcutaneous immunotherapy; WAO, World Allergy Organization.

1 Final visit.

2 One patient had an adverse reaction at the baseline visit that was considered both local and systemic.

## Discussion

Allergen immunotherapy (AIT), by targeting the underlying disease process and inducing long-term desensitization to the relevant allergens, has been demonstrated to be a valuable, disease-modifying treatment option for most patients with cat allergy (29–31). In this retrospective, real-world data study we showed that, in adult patients with moderate to severe allergic rhinitis/rhinoconjunctivitis with or without controlled asthma due to sensitization to cat epithelium, SCIT with Dpg-pol-cat significantly improved rhinitis HRQoL and reduced the presence and severity of clinical symptoms and the use of concomitant rescue medications. In addition, immunotherapy was associated with a significant increase in cat epithelium-specific IgG4 antibody levels. Importantly, Dpg-pol-cat showed a good safety profile, with few systemic reactions, all of grade <2 according to the 2010 WAO grading system, occurring mainly during the maintenance phase. The clinical effectiveness of Dpg-pol-cat was evident after 6 months of treatment and was maintained throughout the study until the final visit at 18/24 months.

Allergic rhinitis and related conditions are recognized as important causes of reduced HRQoL, negatively impacting various aspects of daily life, including physical, emotional, social, and economic aspects, sometimes in a way comparable to more serious medical conditions (32). As with other chronic diseases, the concordance between allergic rhinitis and patient's HRQoL tend to be weak to moderate, highlighting the importance of assessing not only disease severity but also psychosocial factors, in particular patient's ability to cope with their condition (i.e., perceived control), when evaluating the therapeutic effect of the immunotherapy (33, 34). In this regard, rigorous questionnaires applicable to allergic rhinitis have been developed and validated for use in clinical studies (35, 36). We found that, in the context of routine clinical practice, Dpg-pol-cat was associated with a rapid, significant, and sustained improvement in HRQoL from 6 months of treatment, with an overall decrease in questionnaire score of 55.6% from baseline to the LOCF (*p* = 0.0004). Any change in a HRQoL measure has been proposed to be clinically meaningful because it represents the patient's perception of their health burden, but also because HRQoL measures are indicative of the risk/benefit ratio of available treatment options and help to guide patient management (37). SCIT with Dpg-pol-cat was also associated with an overall improvement in disease symptomatology. Most patients who had moderate to severe symptoms at baseline experienced a substantial improvement after treatment and had only mild symptoms or were asymptomatic at the LOCF, particularly for tearing, eye redness and wheezing. In addition, SCIT with Dpg-pol-cat induced progressive asthma control in the subgroup of asthmatic patients as early as 6 months of SCIT, with the proportion of patients with good asthma control almost doubling from baseline to the end of the study. Patients with asthma and severe allergic rhinitis are more likely to have poor asthma control, which is likely to improve with appropriate treatment of the rhinitis (38). The observed reduction in symptomatology was accompanied by a reduction in rescue medication use, with a 50% decrease in dMS recorded at 12 months and maintained until the end of study. These results highlight the role of immunotherapy as a therapeutic option capable of reducing the allergic response without the need for long-term medications that only temporarily mask allergy symptoms and often have unacceptable adverse effects without treating the underlying condition (39, 40). In this line, a systematic review of 88 studies involving 3,459 asthmatic patients exposed to SCIT showed that AIT resulted in a significant reduction in asthma symptoms (i.e., for every 3 patients treated with subcutaneous immunotherapy, 1 case of worsening in asthma symptoms was avoided), medication use (i.e., for every 4 patients treated, 1 patient would avoid an increase in symptomatic medication use) (41).

Another important dimension of SCIT with Dpg-pol-cat in relation to patient's HRQoL is its benefit on the psychologic impact of pet allergy, which can be of great concern, particularly when owners—most of whom feel their cats are members of the family—, are asked to make lifestyle changes involving avoidance of animal ownership. The human-animal bond is recognized by the American Veterinary Medical Association as a mutually beneficial and dynamic relationship between humans and animals that is influenced by behaviours considered essential to the health and well-being of both (42).

The induction of regulatory T cells and the production of allergen-specific IgG4 blocking antibodies has been proposed to be an essential mechanism of immune tolerance to allergens, preventing the activation of these cells (43). Analyzing the immunological response of patients to SCIT with Dpg-pol-cat in our study, we found that the levels of allergen-specific IgG4 antibodies increased significantly from baseline to the last visit from 3.84 to 4.43 kU/L (*p* = 0.0092), most likely reflecting successful desensitization.

Of note, and as reported in previous studies with AIT (29, 44, 45), the safety profile of Dpg-pol-cat during the rush up-dosing and maintenance phases was good, with only 5 systemic reactions reported—2 during the rush built-up phase and 3 during the maintenance phase—, all of which were grade 1 or 2, and resolved without the need for concomitant treatment or treatment adjustment in most patients. Only one patient discontinued the treatment due to adverse reactions. Our results are consistent with those reported from a prospective, multicentre, non-interventional post-authorization safety study conducted in Germany from May 2022 to January 2024, which evaluated the safety of Dpg-pol-cat in a similar population of patients with cat allergy to cat epithelium and allergic rhinitis/rhinoconjunctivitis, with or without controlled asthma (*n* = 100). In this study, no epinephrine was required, and only one patient experienced a serious adverse event after the first injection (i.e., shortness of breath), which resolved within 30 min. In general, most adverse events were delayed local reactions and most systemic reactions were classified as grade 1 (46).

The safety and effectiveness of AIT with Dpg-pol-cat were recently demonstrated in a real-world, retrospective study also conducted in Madrid, Spain, involving 62 patients aged ≥12 years with cat allergy and moderate to severe allergic rhinitis/rhinoconjunctivitis, with or without asthma. The treatment showed a favorable safety profile: 7 patients (10.6%) experienced systemic reactions, all classified as grade 1 according to the EAACI system. Importantly, only 3 patients discontinued AIT, and in all cases, the discontinuation was due to reasons unrelated to the systemic reactions. Regarding effectiveness, treatment with Dpg-pol-cat led to a significant reduction in the proportion of patients with moderate to severe rhinitis at 12 months (from 88.2% to 29.4%; *p* < 0.0001), as well as in those with moderate asthma (from 76.5% to 38.2%; *p* = 0.0004). AIT was also associated with improved pulmonary function, with mean FEV₁ increasing from 3,188.9 ml to 3,419.6 ml (*p* = 0.0023), and a marked reduction in the use of rescue medication for rhinitis (from 94.1% to 23.5%; *p* < 0.0001). Most patients reported improvement in rhinitis (97.1%) and asthma (92.6%) symptoms (31).

Although the effectiveness and safety of AIT have been extensively demonstrated in both clinical trials and routine practice, its success largely depends on adapting treatment to the individual characteristics, needs, and preferences of each patient (47). This is even more important in recent years, when an increasing prevalence of respiratory diseases such as rhinitis, rhinoconjunctivitis and asthma has been observed due to an increase in allergy triggers, mainly related to air pollution and climate change (48, 49). In the context of available therapies for respiratory allergy, AIT is the prototype of current and future precision medicine (50–52). Although validated predictive biomarkers to identify patients who will benefit most from treatment are not yet available (53). In addition, due to its specificity and long-term therapeutic effect, AIT has a major impact on pharmacoeconomic evaluation (54).

This study has several strengths and limitations that should be acknowledged. A key strength is the significant improvement in HRQoL observed among patients receiving SCIT with Dpg-pol-cat, despite the small sample size. High treatment adherence and the use of real-world data derived from routine clinical practice enhance the study's relevance and applicability further. Notably, the research team successfully captured primary endpoint data on HRQoL under the challenging conditions imposed by the pandemic, reflecting their commitment and adaptability. The main limitations were the modest sample size and the lack of a control group. Nevertheless, single-arm studies can offer valuable insights into treatment effects when interpreted in the context of historical or implicit comparators, as prior literature suggests. Additionally, because the study was conducted during the COVID-19 pandemic, as previously mentioned, routine collection of some parameters, including spirometry (i.e., FEV_1_), was interrupted or partially hampered.

## Conclusion

In this real-world evidence study in adult patients sensitized to cat epithelium with moderate rhinoconjunctivitis and asthma, SCIT with Dpg-pol-cat proved to be a valuable, effective and safe therapeutic option. Dpg-pol-cat significantly improved rhinitis HRQoL, reduced rhinoconjunctivitis and asthma symptom severity, and significantly decreased the use of concomitant medications for both rhinitis and asthma. In the subgroup of asthmatic patients, asthma control was also significantly higher after SCIT treatment. In addition, Dpg-pol-cat significantly increased cat epithelium-specific IgG4 antibody levels, highlighting its immunological impact in this patient population.

## Data Availability

The raw data supporting the conclusions of this article will be made available by the authors, without undue reservation.

## References

[1] Bantz SK Zhu Z Zheng T. The atopic march: progression from atopic dermatitis to allergic rhinitis and asthma. J Clin Cell Immunol. (2014) 5(2):202. 10.4172/2155-9899.1000202 25419479 PMC4240310

[2] Pawankar R. Allergic diseases and asthma: a global public health concern and a call to action. World Allergy Organ J. (2014) 7(1):12. 10.1186/1939-4551-7-12 24940476 PMC4045871

[3] Woodfolk JA Commins SP Schuyler AJ Erwin EA Platts-Mills TA. Allergens, sources, particles, and molecules: why do we make IgE responses? Allergol Int. (2015) 64(4):295–303. 10.1016/j.alit.2015.06.001 26433525 PMC5406225

[4] Asher MI Montefort S Björkstén B Lai CK Strachan DP Weiland SK. Worldwide time trends in the prevalence of symptoms of asthma, allergic rhinoconjunctivitis, and eczema in childhood: ISAAC phases one and three repeat multicountry cross-sectional surveys. Lancet. (2006) 368(9537):733–43. 10.1016/s0140-6736(06)69283-0 16935684

[5] Björkstén B Clayton T Ellwood P Stewart A Strachan D. Worldwide time trends for symptoms of rhinitis and conjunctivitis: phase III of the international study of asthma and allergies in childhood. Pediatr Allergy Immunol. (2008) 19(2):110–24. 10.1111/j.1399-3038.2007.00601.x 17651373

[6] Aldakheel FM. Allergic diseases: a comprehensive review on risk factors, immunological mechanisms, link with COVID-19, potential treatments, and role of allergen bioinformatics. Int J Environ Res Public Health. (2021) 18(22):12105. 10.3390/ijerph182212105 34831860 PMC8622387

[7] European Academy of Allergy and Clinical Immunology (EAACI). Global atlas of allergy (2014). Available online at: https://www.eaaci.org/images/Atlas/Global_Atlas_IV_v1.pdf (Accessed April 21, 2025).

[8] Zahradnik E Raulf M. Animal allergens and their presence in the environment. Front Immunol. (2014) 5:76. 10.3389/fimmu.2014.00076 24624129 PMC3939690

[9] Sparkes AH. Human allergy to cats: a review for veterinarians on prevalence, causes, symptoms and control. J Feline Med Surg. (2022) 24(1):31–42. 10.1177/1098612X211036793 34622710 PMC8721530

[10] Zahradnik E Raulf M. Respiratory allergens from furred mammals: environmental and occupational exposure. Vet Sci. (2017) 4(3):38. 10.3390/vetsci4030038 29056697 PMC5644656

[11] Sparkes AH. Human allergy to cats: a review of the impact on cat ownership and relinquishment. J Feline Med Surg. (2022) 24(1):43–52. 10.1177/1098612X211013016 34622709 PMC8721548

[12] Warm K Lindberg A Lundbäck B Rönmark E. Increase in sensitization to common airborne allergens among adults—two population-based studies 15 years apart. Allergy Asthma Clin Immunol. (2013) 9(1):20. 10.1186/1710-1492-9-20 23758681 PMC3684537

[13] Cacheiro-Llaguno C Mösges R Calzada D González-de la Fuente S Quintero E Carnés J. Polysensitisation is associated with more severe symptoms: the reality of patients with allergy. Clin Exp Allergy. (2024) 57(8):607–20. 10.1111/cea.14486 38676405

[14] Sociedad Española de Alergología e Immunology Clínica (SEAIC). Informe alergológica 2015 (2015). Available online at: https://www.seaic.org/inicio/noticias-general/alergologica-2015.html (Accessed April 21, 2025).

[15] World Health Organization and International Union of Immunological Societies (WHO/IUIS). Allergen nomenclature sub committee (2024). Available online at: https://allergen.org (Accessed April 21, 2025).

[16] Bonnet B Messaoudi K Jacomet F Michaud E Fauquert JL Caillaud D. An update on molecular cat allergens: fel d 1 and what else? Chapter 1: fel d 1, the major cat allergen. Allergy Asthma Clin Immunol. (2018) 14:14. 10.1186/s13223-018-0239-8 29643919 PMC5891966

[17] van Ree R van Leeuwen WA Bulder I Bond J Aalberse RC. Purified natural and recombinant fel d 1 and cat albumin in *in vitro* diagnostics for cat allergy. J Allergy Clin Immunol. (1999) 104(6):1223–30. 10.1016/s0091-6749(99)70017-5 10589005

[18] Wood RA Chapman MD Adkinson NF Jr Eggleston PA. The effect of cat removal on allergen content in household-dust samples. J Allergy Clin Immunol. (1989) 83(4):730–4. 10.1016/0091-6749(89)90006-7 2708734

[19] Custovic A Simpson A Pahdi H Green RM Chapman MD Woodcock A. Distribution, aerodynamic characteristics, and removal of the major cat allergen fel d 1 in British homes. Thorax. (1998) 53(1):33–8. 10.1136/thx.53.1.33 9577519 PMC1758692

[20] Ichikawa K Iwasaki E Baba M Chapman MD. High prevalence of sensitization to cat allergen among Japanese children with asthma, living without cats. Clin Exp Allergy. (1999) 29(6):754–61. 10.1046/j.1365-2222.1999.00472.x 10336590

[21] Kucuksezer UC Ozdemir C Cevhertas L Ogulur I Akdis M Akdis CA. Mechanisms of allergen-specific immunotherapy and allergen tolerance. Allergol Int. (2020) 69(4):549–60. 10.1016/j.alit.2020.08.002 32900655

[22] Mosges R Valero Santiago A Allekotte S Jahed N Astvatsatourov A Sager A. Subcutaneous immunotherapy with depigmented-polymerized allergen extracts: a systematic review and meta-analysis. Clin Transl Allergy. (2019) 9(1):29. 10.1186/s13601-019-0268-5 31171962 PMC6549305

[23] Ibarrola I Sanz ML Gamboa PM Mir A Benahmed D Ferrer A. Biological characterization of glutaraldehyde-modified Parietaria judaica pollen extracts. Clin Exp Allergy. (2004) 34(2):303–9. 10.1111/j.1365-2222.2004.01859.x 14987312

[24] Nguyen NT Raskopf E Shah-Hosseini K Zadoyan G Mösges R. A review of allergoid immunotherapy: is cat allergy a suitable target? Immunotherapy. (2016) 8(3):331–49. 10.2217/imt.15.121 26860435

[25] Morales M Gallego M Iraola V Taulés M de Oliveira E Moya R. *In vitro* evidence of efficacy and safety of a polymerized cat dander extract for allergen immunotherapy. BMC Immunol. (2017) 18(1):10. 10.1186/s12865-017-0193-0 28235411 PMC5324274

[26] Laguna-Martínez JJ Jiménez A González-Mendiola R Boteanu C Dionicio A González Cardenosa A, editors. Effectiveness and safety using a depigmented, polymerized extract of cat epithelium: a real-world study [#001451]. European Academy of Allergy and Clinical Immunology Hybrid Congress; Hamburg, Germany: Allergy (9–11 June, 2023).

[27] Brożek JL Bousquet J Agache I Agarwal A Bachert C Bosnic-Anticevich S. Allergic rhinitis and its impact on asthma (ARIA) guidelines-2016 revision. J Allergy Clin Immunol. (2017) 140(4):950–8. 10.1016/j.jaci.2017.03.050 28602936

[28] Mauer Y Taliercio RM. Managing adult asthma: the 2019 GINA guidelines. Cleve Clin J Med. (2020) 87(9):569–75. 10.3949/ccjm.87a.19136 32868307

[29] Uriarte SA Sastre J. Subcutaneous immunotherapy with high-dose cat and dog extracts: a real-life study. J Investig Allergol Clin Immunol. (2020) 30(3):169–74. 10.18176/jiaci.0415 31132032

[30] Dhami S Agarwal A. Does evidence support the use of cat allergen immunotherapy? Curr Opin Allergy Clin Immunol. (2018) 18(4):350–5. 10.1097/aci.0000000000000457 29870462

[31] Vázquez de la Torre M López-González P Haroun-Díaz E Somoza ML Cervera MD Ruiz-García M. Depigmented, polymerized cat epithelium extract is safe and improves rhinitis and asthma symptoms in cat-allergic patients: a real-world retrospective study. Int Arch Allergy Immunol. (2025) 186(6):532–42. 10.1159/000541838 39541958

[32] Chen H Katz PP Eisner MD Yelin EH Blanc PD. Health-related quality of life in adult rhinitis: the role of perceived control of disease. J Allergy Clin Immunol. (2004) 114(4):845–50. 10.1016/j.jaci.2004.07.008 15480325

[33] Thompson AK Juniper E Meltzer EO. Quality of life in patients with allergic rhinitis. Ann Allergy Asthma Immunol. (2000) 85(5):338–47; quiz 47–8. 10.1016/s1081-1206(10)62543-4 11101172

[34] Katz PP Yelin EH Eisner MD Blanc PD. Perceived control of asthma and quality of life among adults with asthma. Ann Allergy Asthma Immunol. (2002) 89(3):251–8. 10.1016/s1081-1206(10)61951-5 12269644

[35] Valero A Alonso J Antepara I Baró E Colas C del Cuvillo A. Development and validation of a new Spanish instrument to measure health-related quality of life in patients with allergic rhinitis: the ESPRINT questionnaire. Value Health. (2007) 10(6):466–77. 10.1111/j.1524-4733.2007.00202.x 17970929

[36] Valero A Alonso J Antepara I Baro E Colas C del Cuvillo A. Health-related quality of life in allergic rhinitis: comparing the short form ESPRINT-15 and MiniRQLQ questionnaires. Allergy. (2007) 62(12):1372–8. 10.1111/j.1398-9995.2007.01552.x 17983372

[37] Lydick E Epstein RS. Interpretation of quality of life changes. Qual Life Res. (1993) 2(3):221–6. 10.1007/bf00435226 8401458

[38] Clatworthy J Price D Ryan D Haughney J Horne R. The value of self-report assessment of adherence, rhinitis and smoking in relation to asthma control. Prim Care Respir J. (2009) 18(4):300–5. 10.4104/pcrj.2009.00037 19562233 PMC6619365

[39] Cox L Nelson H Lockey R Calabria C Chacko T Finegold I. Allergen immunotherapy: a practice parameter third update. J Allergy Clin Immunol. (2011) 127(1 Suppl):S1–55. 10.1016/j.jaci.2010.09.034 21122901

[40] Clark J White ND. Immunotherapy for cat allergies: a potential strategy to scratch back. Am J Lifestyle Med. (2017) 11(4):310–3. 10.1177/1559827617701389 30202348 PMC6125099

[41] Abramson MJ Puy RM Weiner JM. Injection allergen immunotherapy for asthma. Cochrane Database Syst Rev. (2010) 8: Cd001186. 10.1002/14651858.CD001186.pub2 PMC13199908 20687065

[42] American Veterinary Medical Association. Human-animal bond. Available online at: https://www.avma.org/resources-tools/one-health/human-animal-bond#:∼:text=The%20human%2Danimal%20bond%20is,wellbeing%20of%20people%20and%20animals (Accessed April 21, 2025).

[43] Durham SR Shamji MH. Allergen immunotherapy: past, present and future. Nat Rev Immunol. (2023) 23(5):317–28. 10.1038/s41577-022-00786-1 36253555 PMC9575636

[44] Calderón MA Vidal C Rodríguez Del Río P Just J Pfaar O Tabar AI. European Survey on adverse systemic reactions in allergen immunotherapy (EASSI): a real-life clinical assessment. Allergy. (2017) 72(3):462–72. 10.1111/all.13066 27718250

[45] Coutinho C Lourenço T Fernandes M Neto M Lopes A Spínola Santos A. Subcutaneous immunotherapy with aeroallergens: safety profile assessment. Eur Ann Allergy Clin Immunol. (2022) 54(2):77–83. 10.23822/EurAnnACI.1764-1489.194 33728836

[46] Mösges R Neumeyr D Weber J Sager A, editors. Subcutaneous immunotherapy with a depigmented cat allergoid in patients with cat allergy is safe and results primarily in delayed local reactions [D3.209]. European Academy of Allergy and Clinical Immunology (EAACI) Congress 2024; 31 May–03 June 2024; Valencia, Spain (2024).

[47] Hamburg MA Collins FS. The path to personalized medicine. N Engl J Med. (2010) 363(4):301–4. 10.1056/NEJMp1006304 20551152

[48] D'Amato G Vitale C Lanza M Molino A D'Amato M. Climate change, air pollution, and allergic respiratory diseases: an update. Curr Opin Allergy Clin Immunol. (2016) 16(5):434–40. 10.1097/aci.0000000000000301 27518837

[49] Urrutia-Pereira M Guidos-Fogelbach G Solé D. Climate changes, air pollution and allergic diseases in childhood and adolescence. J Pediatr (Rio J). (2022) 98(Suppl 1):S47–54. 10.1016/j.jped.2021.10.005 34896064 PMC9510908

[50] Canonica GW Bachert C Hellings P Ryan D Valovirta E Wickman M. Allergen immunotherapy (AIT): a prototype of precision medicine. World Allergy Organ J. (2015) 8(1):31. 10.1186/s40413-015-0079-7 26594303 PMC4640346

[51] Incorvaia C Ridolo E Bagnasco D Scurati S Canonica GW. Personalized medicine and allergen immunotherapy: the beginning of a new era? Clin Mol Allergy. (2021) 19(1):10. 10.1186/s12948-021-00150-z 34233706 PMC8262025

[52] Zemelka-Wiacek M Agache I Akdis CA Akdis M Casale TB Dramburg S. Hot topics in allergen immunotherapy, 2023: current status and future perspective. Allergy. (2023) 79:823–42. 10.1111/all.15945 37984449

[53] López JF Imam B Satitsuksanoa M Lems P Yang S Hwang M. Mechanisms and biomarkers of successful allergen-specific immunotherapy. Asia Pac Allergy. (2022) 12(4):e45. 10.5415/apallergy.2022.12.e45 36452016 PMC9669467

[54] Passalacqua G Canonica GW. AIT (Allergen immunotherapy): a model for the “precision medicine”. Clin Mol Allergy. (2015) 13:24. 10.1186/s12948-015-0028-6 26451132 PMC4597399

