# Correction: Effectiveness and safety of subcutaneous immunotherapy using a depigmented, polymerized extract of cat epithelium in allergic patients: a retrospective, real-world study

**DOI:** 10.3389/falgy.2026.1918161

**Published:** 2026-09-02

**Authors:** María Aránzazu Jiménez-Blanco, María Rosario González-Mendiola, Cosmin Boteanu, Javier Dionicio Elera, Maria Luisa Sánchez-Millán, M. Ruíz-García, Jose Julio Laguna

**Affiliations:** 1 Allergy-Anesthesia Unit, Allergy Department, Hospital Central de la Cruz Roja San José y Santa Adela, Madrid, Spain; 2 Medical Affairs Unit, LETI Pharma, S.L.U., Madrid, Spain; 3 Faculty of Medicine, Universidad Alfonso X El Sabio, Madrid, Spain

**Keywords:** allergen immunotherapy, allergic asthma, allergic rhinitis/rhinoconjunctivitis, cat allergen extract, real-world evidence

There was a mistake in Figure 1 as published. The image for Figure 1 was inadvertently swapped with Figure 2. The captions were correct.

The correct Figure 1 and its caption appears below:

**Figure 1:**
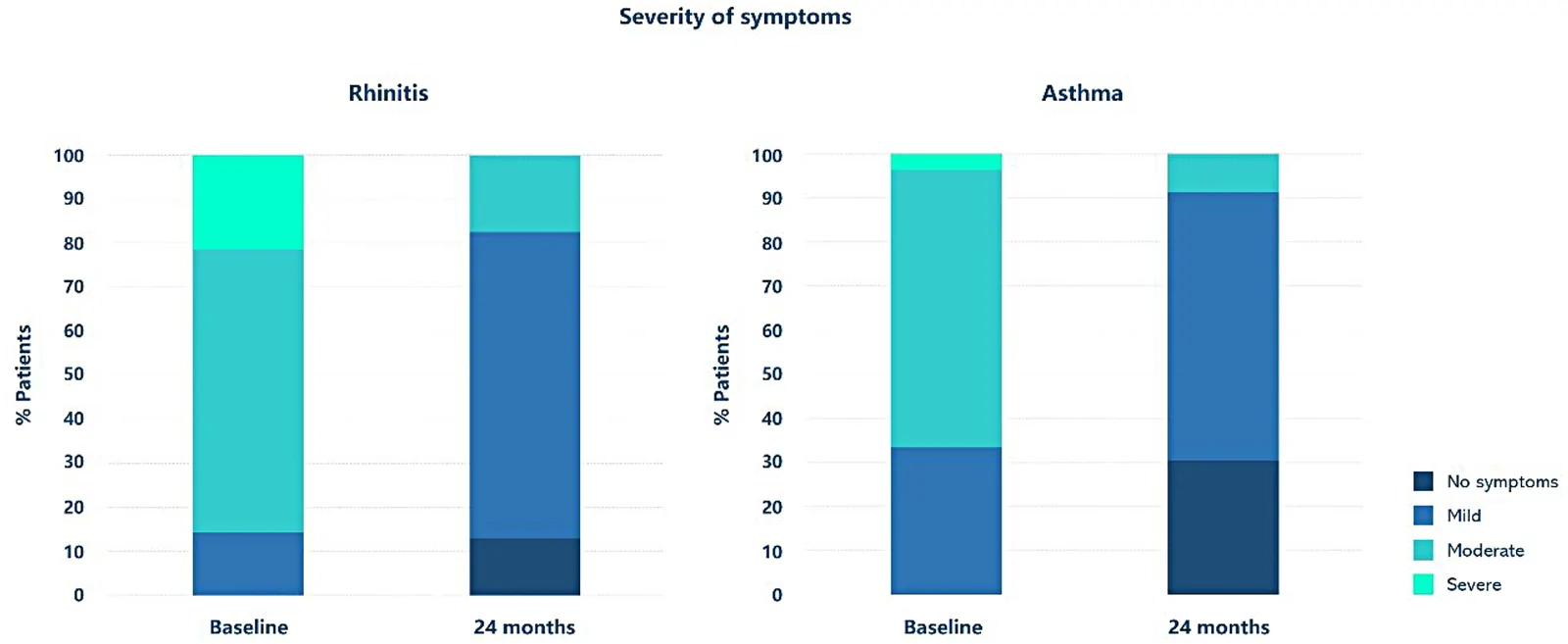


**Figure 2:**
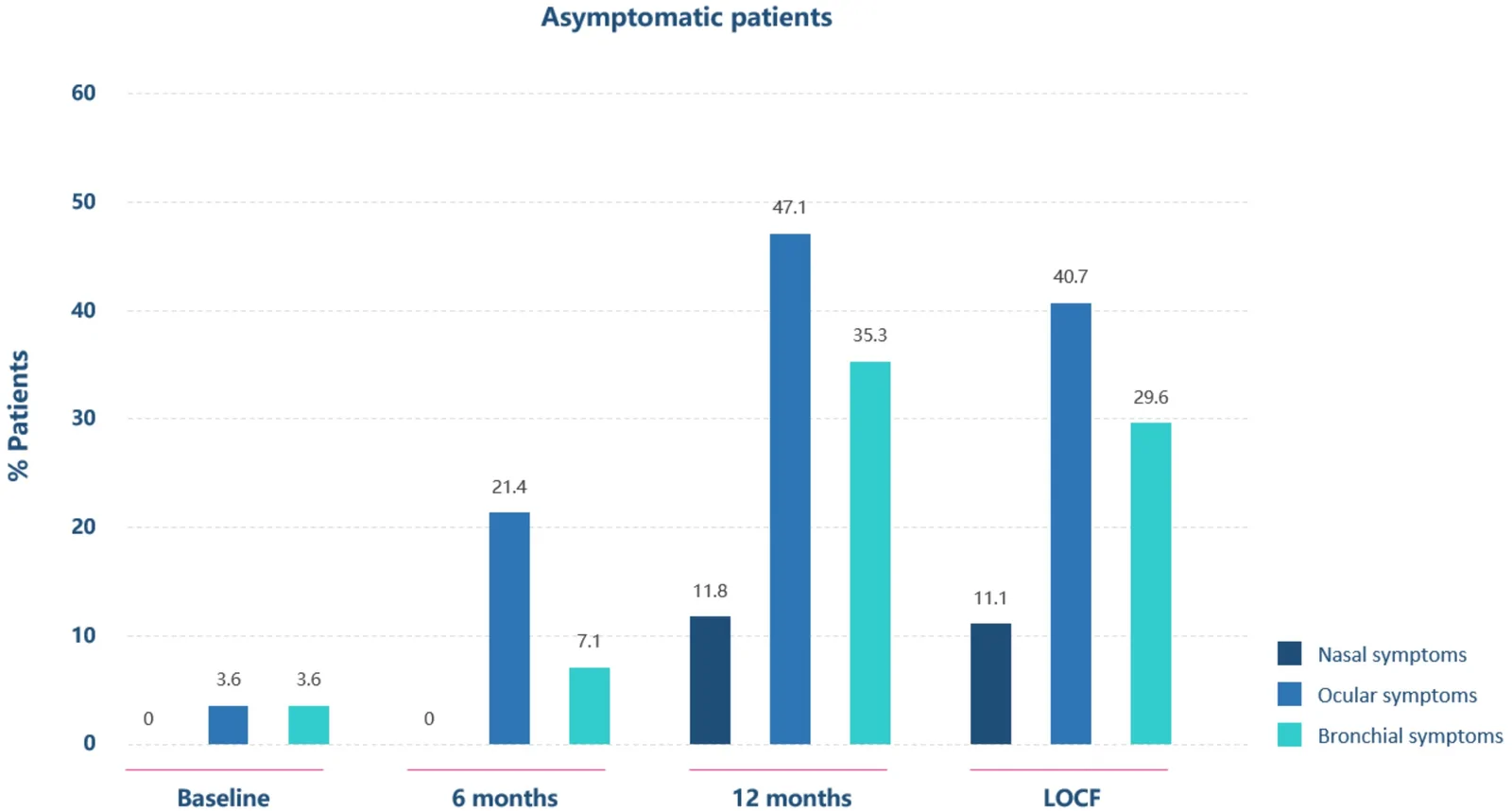


**Figure 1**. Percentage of patients with different severity of rhinitis (left panel) and asthma (right panel) symptoms at baseline and after 24 months of allergen-specific immunotherapy. Symptom severity is color-coded as shown in the box at the bottom right of the figure.

The corrected Figure 2 and its caption appears below:

**Figure 2**. Evolution of the percentage of patients without nasal, ocular and bronchial symptoms at baseline and after 6 months, 12 months and LOCF (Last Observation Carried Forward) of allergen-specific immunotherapy. Percentages are shown above each histogram bar. Symptoms are color-coded as shown in the box at the bottom right of the figure.

The original version of this article has been updated.

