# Supplementary material for: Effectiveness and safety of subcutaneous immunotherapy using a depigmented, polymerized extract of cat epithelium in allergic patients: a retrospective, real-world study
Source: Front Allergy. 2025 Sep 18;6:1642315. doi: 10.3389/falgy.2025.1642315 (PMC12488638; doi:10.3389/falgy.2025.1642315)
Supplement: Supplementary file 1 [file Table1.docx]

| **Supplementary Table S1. Allergen sensitization** | |
| --- | --- |
| **Variable** | **N = 28** |
| Sensitization to animal’s dander, n (%)^1^ |  |
| Cat | 28 (100.0) |
| Dog | 17 (60.7) |
| Sensitization to house dust mites, n (%) |  |
| All species | 4 (14.3) |
| Dermatophagoides farinae | 4 (14.3) |
| Dermatophagoides pteronyssinus | 4 (14.3) |
| Sensitization to moulds, n (%) |  |
| All species | 2 (7.1) |
| *Alternaria alternata* | 2 (7.1) |
| *Aspergillus fumigatus* | 0 (0.0) |
| *Cladosporium herbarum* | 0 (0.0) |
| Sensitization to pollen, n (%) |  |
| All species | 21 (75.0) |
| *Lolium perenne* | 17 (60.7) |
| *Phleum pratense* | 17 (60.7) |
| *Cynodon dactylon* | 9 (32.1) |
| *Olea europaea* | 15 (53.6) |
| Ash tree | 14 (50.0) |
| *Cupressus arizonica* | 12 (42.9) |
| *Platanus acerifolia* | 5 (17.9) |
| *Plantago lanceolata* | 10 (35.7) |
| *Chenopodium album* | 4 (14.3) |
| *Salsola Kali* | 3 (10.7) |
| *Artemisia vulgaris* | 3 (10.7) |
| Sensitization to panallergens, n (%) |  |
| Total | 2 (7.1) |
| Pho d 12 – Profilin | 1 (3.6) |
| Pru p 3 – LTP | 1 (3.6) |
| Controls, n (%) |  |
| Histamine | 28 (100.0) |
| Physiological saline solution | 0 (0.0) |
| ^1^Allergen sensitization determined by skin prick test.  Abbreviations: LTP, lipid transfer protein. | |
