# Supplementary material for: Effectiveness and safety of subcutaneous immunotherapy using a depigmented, polymerized extract of cat epithelium in allergic patients: a retrospective, real-world study
Source: Front Allergy. 2025 Sep 18;6:1642315. doi: 10.3389/falgy.2025.1642315 (PMC12488638; doi:10.3389/falgy.2025.1642315)
Supplement: Supplementary file 2 [file Table2.docx]

| **Supplementary Table S2. Severity of symptoms** | | | | | | | | | |
| --- | --- | --- | --- | --- | --- | --- | --- | --- | --- |
|  | **Visit** | | | | | | | | |
| **Variable** | **Baseline** | **6 months** | p-value^1^ | **12 months** | p-value | **18/24 months**^2^ | p-value | **LOCF^3^** | p-value |
| Rhinitis |  |  | 0.0209 |  | 0.6638 |  | 0.7576 |  | 0.5307 |
| Total, n (%) | 28 (100.0) | 14 (100.0) |  | 17 (100.0) |  | 23 (100.0) |  | 27 (100.0) |  |
| Asymptomatic, n (%) | 0 (0.0) | 0 (0.0) |  | 2 (11.8) |  | 3 (13.0) |  | 3 (11.1) |  |
| Mild, n (%) | 4 (14.3) | 7 (50.0) |  | 10 (58.8) |  | 16 (69.6) |  | 19 (70.4) |  |
| Moderate, n (%) | 18 (64.3) | 4 (28.6) |  | 4 (23.5) |  | 4 (17.4) |  | 5 (18.5) |  |
| Severe, n (%) | 6 (21.4) | 3 (21.4) |  | 1 (5.9) |  | 0 (0.0) |  | 0 (0.0) |  |
| Missing, n | 0 | 14 |  | 11 |  | 5 |  | 1 |  |
| Rhinorrhea |  |  | 0.0294 |  | 0.3238 |  | 0.1483 |  | 0.4261 |
| Total, n (%) | 28 (100.0) | 14 (100.0) |  | 17 (100.0) |  | 23 (100.0) |  | 27 (100.0) |  |
| Asymptomatic, n (%) | 0 (0.0) | 1 (7.1) |  | 4 (23.5) |  | 8 (34.8) |  | 9 (33.3) |  |
| Mild, n (%) | 5 (17.9) | 7 (50.0) |  | 8 (47.1) |  | 11 (47.8) |  | 13 (48.1) |  |
| Moderate, n (%) | 19 (67.9) | 4 (28.6) |  | 5 (29.4) |  | 4 (17.4) |  | 5 (18.5) |  |
| Severe, n (%) | 4 (14.3) | 2 (14.3) |  | 0 (0.0) |  | 0 (0.0) |  | 0 (0.0) |  |
| Missing, n | 0 | 14 |  | 11 |  | 5 |  | 1 |  |
| Nasal itchiness |  |  | 0.0566 |  | 0.3695 |  | 0.4777 |  | 0.5731 |
| Total, n (%) | 28 (100.0) | 14 (100.0) |  | 17 (100.0) |  | 23 (100.0) |  | 27 (100.0) |  |
| Asymptomatic, n (%) | 0 (0.0) | 1 (7.1) |  | 4 (23.5) |  | 7 (30.4) |  | 8 (29.6) |  |
| Mild, n (%) | 8 (28.6) | 8 (57.1) |  | 11 (64.7) |  | 12 (52.2) |  | 14 (51.9) |  |
| Moderate, n (%) | 17 (60.7) | 4 (28.6) |  | 2 (11.8) |  | 4 (17.4) |  | 5 (18.5) |  |
| Severe, n (%) | 3 (10.7) | 1 (7.1) |  | 0 (0.0) |  | 0 (0.0) |  | 0 (0.0) |  |
| Missing, n | 0 | 14 |  | 11 |  | 5 |  | 1 |  |
| Nasal obstruction |  |  | 0.0205 |  | 0.0518 |  | 0.8543 |  | 0.7337 |
| Total, n (%) | 28 (100.0) | 14 (100.0) |  | 17 (100.0) |  | 23 (100.0) |  | 27 (100.0) |  |
| Asymptomatic, n (%) | 1 (3.6) | 2 (14.3) |  | 9 (52.9) |  | 10 (43.5) |  | 11 (40.7) |  |
| Mild, n (%) | 7 (25.0) | 6 (42.9) |  | 3 (17.6) |  | 9 (39.1) |  | 11 (40.7) |  |
| Moderate, n (%) | 13 (46.4) | 3 (21.4) |  | 4 (23.5) |  | 4 (17.4) |  | 5 (18.5) |  |
| Severe, n (%) | 7 (25.0) | 3 (21.4) |  | 1 (5.9) |  | 0 (0.0) |  | 0 (0.0) |  |
| Missing, n | 0 | 14 |  | 11 |  | 5 |  | 1 |  |
| Conjunctivitis |  |  | 0.1051 |  | 0.4682 |  | 0.4926 |  | 0.7129 |
| Total, n (%) | 28 (100.0) | 14 (100.0) |  | 17 (100.0) |  | 23 (100.0) |  | 27 (100.0) |  |
| Asymptomatic, n (%) | 1 (3.6) | 4 (28.6) |  | 8 (47.1) |  | 12 (52.2) |  | 14 (51.9) |  |
| Mild, n (%) | 14 (50.0) | 8 (57.1) |  | 8 (47.1) |  | 11 (47.8) |  | 12 (44.4) |  |
| Moderate, n (%) | 12 (42.9) | 2 (14.3) |  | 0 (0.0) |  | 0 (0.0) |  | 0 (0.0) |  |
| Severe, n (%) | 1 (3.6) | 0 (0.0) |  | 1 (5.9) |  | 0 (0.0) |  | 1 (3.7) |  |
| Missing, n | 0 | 14 |  | 11 |  | 5 |  | 1 |  |
| Eye itchiness |  |  | 0.3891 |  | 0.5710 |  | 0.4157 |  | 0.8023 |
| Total, n (%) | 28 (100.0) | 14 (100.0) |  | 17 (100.0) |  | 23 (100.0) |  | 27 (100.0) |  |
| Asymptomatic, n (%) | 1 (3.6) | 3 (21.4) |  | 8 (47.1) |  | 9 (39.1) |  | 11 (40.7) |  |
| Mild, n (%) | 12 (42.9) | 8 (57.1) |  | 8 (47.1) |  | 14 (60.9) |  | 15 (55.6) |  |
| Moderate, n (%) | 12 (42.9) | 3 (21.4) |  | 0 (0.0) |  | 0 (0.0) |  | 0 (0.0) |  |
| Severe, n (%) | 3 (10.7) | 0 (0.0) |  | 1 (5.9) |  | 0 (0.0) |  | 1 (3.7) |  |
| Missing, n | 0 | 14 |  | 11 |  | 5 |  | 1 |  |
| Tearing |  |  | 0.0168 |  | 0.1282 |  | 0.0539 |  | 0.1112 |
| Total, n (%) | 28 (100.0) | 14 (100.0) |  | 17 (100.0) |  | 23 (100.0) |  | 27 (100.0) |  |
| Asymptomatic, n (%) | 5 (17.9) | 7 (50.0) |  | 13 (76.5) |  | 14 (60.9) |  | 17 (63.0) |  |
| Mild, n (%) | 15 (53.6) | 6 (42.9) |  | 3 (17.6) |  | 9 (39.1) |  | 9 (33.3) |  |
| Moderate, n (%) | 7 (25.0) | 1 (7.1) |  | 1 (5.9) |  | 0 (0.0) |  | 1 (3.7) |  |
| Severe, n (%) | 1 (3.6) | 0 (0.0) |  | 0 (0.0) |  | 0 (0.0) |  | 0 (0.0) |  |
| Missing, n | 0 | 14 |  | 11 |  | 5 |  | 1 |  |
| Eye redness (erythema) |  |  | 0.0239 |  | 0.1981 |  | 0.2214 |  | 0.2419 |
| Total, n (%) | 28 (100.0) | 14 (100.0) |  | 17 (100.0) |  | 23 (100.0) |  | 27 (100.0) |  |
| Asymptomatic, n (%) | 12 (42.9) | 12 (85.7) |  | 15 (88.2) |  | 20 (87.0) |  | 24 (88.9) |  |
| Mild, n (%) | 11 (39.3) | 2 (14.3) |  | 2 (11.8) |  | 3 (13.0) |  | 3 (11.1) |  |
| Moderate, n (%) | 5 (17.9) | 0 (0.0) |  | 0 (0.0) |  | 0 (0.0) |  | 0 (0.0) |  |
| Severe, n (%) | 0 (0.0) | 0 (0.0) |  | 0 (0.0) |  | 0 (0.0) |  | 0 (0.0) |  |
| Missing, n | 0 | 14 |  | 11 |  | 5 |  | 1 |  |
| Asthma |  |  | 0.196 |  | 0.493 |  | 0.662 |  | 0.662 |
| Total, n (%) | 27 (100.0) | 14 (100.0) |  | 16 (100.0) |  | 23 (100.0) |  | 26 (100.0) |  |
| Asymptomatic, n (%) | 1 (3.7) | 2 (14.3) |  | 4 (25.0) |  | 7 (30.4) |  | 7 (26.9) |  |
| Mild, n (%) | 8 (29.6) | 7 (50.0) |  | 9 (56.3) |  | 14 (60.9) |  | 17 (65.4) |  |
| Moderate, n (%) | 17 (63.0) | 5 (35.7) |  | 3 (18.8) |  | 2 (8.7) |  | 2 (7.7) |  |
| Severe, n (%) | 1 (3.7) | 0 (0.0) |  | 0 (0.0) |  | 0 (0.0) |  | 0 (0.0) |  |
| Missing, n | 0 | 13 |  | 11 |  | 4 |  | 4 |  |
| Cough |  |  | 0.349 |  | 0.411 |  | 0.281 |  | 0.2811 |
| Total, n (%) | 27 (100.0) | 14 (100.0) |  | 16 (100.0) |  | 23 (100.0) |  | 27 (100.0) |  |
| Asymptomatic, n (%) | 2 (7.4) | 6 (42.9) |  | 11 (68.8) |  | 11 (47.8) |  | 15 (55.6) |  |
| Mild, n (%) | 9 (55.6) | 7 (50.0) |  | 5 (31.3) |  | 12 (52.2) |  | 12 (44.4) |  |
| Moderate, n (%) | 10 (37.0) | 1 (7.1) |  | 0 (0.0) |  | 0 (0.0) |  | 0 (0.0) |  |
| Severe, n (%) | 0 (0.0) | 0 (0.0) |  | 0 (0.0) |  | 0 (0.0) |  | 0 (0.0) |  |
| Missing, n | 0 | 13 |  | 11 |  | 4 |  | 0 |  |
| Dyspnea |  |  | 0.464 |  | 0.274 |  | 0.401 |  | 0.4013 |
| Total, n (%) | 27 (100.0) | 14 (100.0) |  | 16 (100.0) |  | 23 (100.0) |  | 26 (100.0) |  |
| Asymptomatic, n (%) | 1(3.7) | 3 (21.4) |  | 8 (50.0) |  | 7 (30.4) |  | 11 (42.3) |  |
| Mild, n (%) | 7 (25.9) | 5 (35.7) |  | 5 (31.3) |  | 14 (60.9) |  | 14 (53.9) |  |
| Moderate, n (%) | 18 (66.6) | 6 (42.9) |  | 3 (17.7) |  | 2 (8.7) |  | 1 (3.9) |  |
| Severe, n (%) | 1 (3.7) | 0 (0.0) |  | 0 (0.0) |  | 0 (0.0) |  | 0 (0.0) |  |
| Missing, n | 0 | 13 |  | 11 |  | 4 |  | 1 |  |
| Wheezing |  |  | 0.001 |  | 0.619 |  | 0.042 |  | 0.0416 |
| Total, n (%) | 27 (100.0) | 14 (100.0) |  | 17 (100.0) |  | 23 (100.0) |  | 27 (100.0) |  |
| Asymptomatic, n (%) | 7 (26.0) | 6 (42.9) |  | 11 (64.7) |  | 16 (69.6) |  | 19 (70.4) |  |
| Mild, n (%) | 10 (37.0) | 7 (50.0 |  | 4 (23.5) |  | 7 (30.4) |  | 8 (29.6) |  |
| Moderate, n (%) | 9 (33.0) | 1 (7.1) |  | 2 (11.8) |  | 0 (0.0) |  | 0 (0.0) |  |
| Severe, n (%) | 1 (4.0) | 0 (0.0) |  | 0 (0.0) |  | 0 (0.0) |  | 0 (0.0) |  |
| Missing, n | 0 | 13 |  | 10 |  | 4 |  | 0 |  |
| ^1^Chi-square test; ^2^Final visit; ^3^If information from the last visit is missing, the same information from the 12-month visit is assigned to the same patient.  Abbreviations: N/A, not applicable; LOCF, last observation carried forward. | | | | | | | | | |
|  |  |  |  |  |  |  |  |  |  |
