# Supplementary material for: Effectiveness and safety of subcutaneous immunotherapy using a depigmented, polymerized extract of cat epithelium in allergic patients: a retrospective, real-world study
Source: Front Allergy. 2025 Sep 18;6:1642315. doi: 10.3389/falgy.2025.1642315 (PMC12488638; doi:10.3389/falgy.2025.1642315)
Supplement: Supplementary file 3 [file Table3.docx]

| **Supplementary Table S3. Symptom improvement** | | | | | | | | |
| --- | --- | --- | --- | --- | --- | --- | --- | --- |
| **Variable** | **6 months** | p-value^1^ | **12 months** | p-value | **18/24 months^2^** | p-value | **Final LOCF^3^** | p-value |
| Rhinorrhea |  | n.d.^4^ |  | 0.285 |  | 0.416 |  | 0.515 |
| Total, n (%) | 14 (100.0) |  | 17 (100.0) |  | 23 (100.0) |  | 27 (100.0) |  |
| Improvement, n (%) | 7 (50.0) |  | 12 (70.6) |  | 19 (82.6) |  | 21 (77.8) |  |
| No improvement, n (%) | 7 (50.0) |  | 5 (29.4) |  | 4 (17.4) |  | 6 (22.2) |  |
| Missing, n | 14 |  | 11 |  | 5 |  | 1 |  |
| Nasal itchiness |  | n.d. |  | 0.064 |  | 0.164 |  | 0.124 |
| Total, n (%) | 14 (100.0) |  | 17 (100.0) |  | 23 (100.0) |  | 27 (100.0) |  |
| Improvement, n (%) | 8 (57.1) |  | 14 (82.4) |  | 17 (73.9) |  | 20 (74.1) |  |
| No improvement, n (%) | 6 (42.9) |  | 3 (17.6) |  | 6 (26.1) |  | 7 (25.9) |  |
| Missing, n | 14 |  | 11 |  | 5 |  | 1 |  |
| Nasal obstruction |  | n.d. |  | n.d. |  | 0.057 |  | 0.078 |
| Total, n (%) | 14 (100.0) |  | 17 (100.0) |  | 23 (100.0) |  | 27 (100.0) |  |
| Improvement, n (%) | 5 (35.7) |  | 14 (82.4) |  | 17 (73.9) |  | 19 (70.4) |  |
| No improvement, n (%) | 9 (64.3) |  | 3 (17.6) |  | 6 (26.1) |  | 8 (29.6) |  |
| Missing, n | 14 |  | 11 |  | 5 |  | 1 |  |
| Eye itchiness |  | n.d. |  | 0.168 |  | 0.135 |  | 0.124 |
| Total, n (%) | 14 (100.0) |  | 17 (100.0) |  | 23 (100.0) |  | 27 (100.0) |  |
| Improvement, n (%) | 7 (50.0) |  | 11 (64.7) |  | 16 (69.6) |  | 18 (66.7) |  |
| No improvement, n (%) | 7 (50.0) |  | 6 (35.3) |  | 7 (30.4) |  | 9 (33.3) |  |
| Missing, n | 14 |  | 11 |  | 5 |  | 1 |  |
| Tearing |  | n.d. |  | 0.028 |  | 0.048 |  | 0.031 |
| Total, n (%) | 14 (100.0) |  | 17 (100.0) |  | 23 (100.0) |  | 27 (100.0) |  |
| Improvement, n (%) | 7 (50.0) |  | 9 (52.9) |  | 16 (69.6) |  | 17 (63.0) |  |
| No improvement, n (%) | 7 (50.0) |  | 8 (47.1) |  | 7 (30.4) |  | 10 (37.0) |  |
| Missing, n | 14 |  | 11 |  | 5 |  | 1 |  |
| Eye redness (Erythema) |  | n.d. |  | 0.028 |  | <0.001 |  | 0.001 |
| Total, n (%) | 14 (100.0) |  | 17 (100.0) |  | 23 (100.0) |  | 27 (100.0) |  |
| Improvement, n (%) | 5 (35.7) |  | 8 (47.1) |  | 11 (47.8) |  | 13 (48.1) |  |
| No improvement, n (%) | 9 (64.3) |  | 9 (52.9) |  | 12 (52.2) |  | 14 (51.9) |  |
| Missing, n | 14 |  | 11 |  | 5 |  | 1 |  |
| Cough |  | n.d. |  | 0.168 |  | 0.135 |  | 0.094 |
| Total, n (%) | 14 (100.0) |  | 17 (100.0) |  | 23 (100.0) |  | 27 (100.0) |  |
| Improvement, n (%) | 7 (50.0) |  | 14 (82.4) |  | 14 (60.9) |  | 18 (66.7) |  |
| No improvement, n (%) | 7 (50.0) |  | 3 (17.6) |  | 9 (39.1) |  | 9 (33.3) |  |
| Missing, n | 14 |  | 11 |  | 5 |  | 1 |  |
| Dyspnea |  | n.d. |  | 0.168 |  | 0.067 |  | 0.051 |
| Total, n (%) | 14 (100.0) |  | 17 (100.0) |  | 23 (100.0) |  | 27 (100.0) |  |
| Improvement, n (%) | 7 (50.0) |  | 12 (70.6) |  | 17 (73.9) |  | 19 (70.4) |  |
| No improvement, n (%) | 7 (50.0) |  | 5 (29.4) |  | 6 (26.1) |  | 8 (29.6) |  |
| Missing, n | 14 |  | 11 |  | 5 |  | 1 |  |
| Wheezes |  | n.d. |  | n.d. |  | 0.009 |  | 0.006 |
| Total, n (%) | 14 (100.0) |  | 17 (100.0) |  | 23 (100.0) |  | 27 (100.0) |  |
| Improvement, n (%) | 8 (57.1) |  | 12 (70.6) |  | 17 (73.9) |  | 19 (70.4) |  |
| No improvement, n (%) | 6 (42.9) |  | 5 (29.4) |  | 6 (26.1) |  | 8 (29.6) |  |
| Missing, n | 14 |  | 11 |  | 5 |  | 1 |  |
| The improvement of each symptom is determined by the decrease in the severity of the symptom.  ^1^Chi-square test; ^2^Final visit; ^3^If information from the last visit was missing, the same information from the 12-month visit was assigned to the same patient; ^4^Calculation of *p*-value was not possible due to missing values in a category.  Abbreviations: LOCF, last observation carried forward; n.d., not determined. | | | | | | | | |
|  |  |  |  |  |  |  |  |  |
