# Supplementary material for: Effectiveness and safety of subcutaneous immunotherapy using a depigmented, polymerized extract of cat epithelium in allergic patients: a retrospective, real-world study
Source: Front Allergy. 2025 Sep 18;6:1642315. doi: 10.3389/falgy.2025.1642315 (PMC12488638; doi:10.3389/falgy.2025.1642315)
Supplement: Supplementary file 4 [file Table4.docx]

| **Supplementary Table S4. Concomitant medications** | | | | | | | | | |
| --- | --- | --- | --- | --- | --- | --- | --- | --- | --- |
|  | **Visit** | | | | | | | | |
| **Variable, n (%)** | **Baseline** | **6 months** | p-value^1^ | **12 months** | p-value | **18/24 months^2^** | p-value | **Final LOCF^3^** | p-value |
| Medication |  |  | n.d.^4^ |  | n.d. |  | n.d. |  | n.d. |
| Total | 28 (100.0) | 28 (100.0) |  | 28 (100.0) |  | 28 (100.0) |  | 28 (100.0) |  |
| Yes | 28 (100.0) | 28 (100.0) |  | 26 (92.9) |  | 25 (89.3) |  | 25 (89.3) |  |
| No | 0 (0.0) | 0 (0.0) |  | 2 (7.1) |  | 3 (10.7) |  | 3 (10.7) |  |
| Missing | 0 | 0 |  | 0 |  | 0 |  | 0 |  |
| Antihistamines |  |  | 0.033 |  | 0.0626 |  | 0.161 |  | 0.161 |
| Total | 28 (100.0) | 28 (100.0) |  | 26 (100.0) |  | 26 (100.0) |  | 26 (100.0) |  |
| Yes | 27 (96.4%) | 25 (89.3) |  | 20 (76.9) |  | 17 (65.4) |  | 17 (65.4) |  |
| No | 1 (3.6%) | 3 (10.7) |  | 6 (23.1) |  | 9 (34.6) |  | 9 (34.6) |  |
| Missing | 0 | 0 |  | 2 |  | 2 |  | 2 |  |
| Antileukotrienes |  |  | <0.0001 |  | <0.0001 |  | 0.0015 |  | 0.0015 |
| Total | 28 (100.0) | 27 (100.0) |  | 27 (100.0) |  | 28 (100.0) |  | 28 (100.0) |  |
| Yes | 7 (25.0) | 6 (22.2) |  | 6 (22.2) |  | 3 (10.7) |  | 3 (10.7) |  |
| No | 21 (75.0) | 21 (77.8) |  | 21 (77.8) |  | 25 (89.3) |  | 25 (89.3) |  |
| Missing | 0 | 1 |  | 1 |  | 0 |  | 0 |  |
| SABAs |  |  | <0.0001 |  | <0.0001 |  | <0.0001 |  | <0.0001 |
| Total | 28 (100.0) | 28 (100.0) |  | 28 (100.0) |  | 28 (100.0) |  | 28 (100.0) |  |
| Yes | 19 (67.9) | 19 (67.9) |  | 18 (64.3) |  | 18 (64.3) |  | 18 (64.3) |  |
| No | 9 (32.1) | 9 (32.1) |  | 10 (35.7) |  | 10 (35.7) |  | 10 (35.7) |  |
| Missing | 0 | 0 |  | 0 |  | 0 |  | 0 |  |
| Nasal CS |  |  | <0.0001 |  | <0.0001 |  | 0.0008 |  | 0.0202 |
| Total | 28 (100.0) | 23 (100.0) |  | 22 (100.0) |  | 22 (100.0) |  | 22 (100.0) |  |
| Yes | 23 (82.1) | 18 (78.3) |  | 14 (63.6) |  | 10 (45.5) |  | 10 (45.5) |  |
| No | 5 (17.9) | 5 (21.7) |  | 8 (36.4) |  | 12 (54.5) |  | 12 (54.5) |  |
| Missing | 0 | 5 |  | 6 |  | 6 |  | 6 |  |
| Oral CS |  |  | n.d. |  | n.d. |  | n.d. |  | n.d. |
| Total | 28 (100.0) | 28 (100.0) |  | 28 (100.0) |  | 28 (100.0) |  | 28 (100.0) |  |
| Yes | 0 (0.0) | 0 (0.0) |  | 0 (0.0) |  | 0 (0.0) |  | 0 (0.0) |  |
| No | 28 (100.0) | 28 (100.0) |  | 28 (100.0) |  | 28 (100.0) |  | 28 (100.0) |  |
| Missing | 0 | 0 |  | 0 |  | 0 |  | 0 |  |
| Nasal CS + Antihistamines |  |  | n.d. |  | n.d. |  | n.d. |  | n.d. |
| Total | 28 (100.0) | 28 (100.0) |  | 28 (100.0) |  | 28 (100.0) |  | 28 (100.0) |  |
| Yes | 3 (10.7) | 3 (10.7) |  | 3 (10.7) |  | 3 (10.7) |  | 3 (10.7) |  |
| No | 25 (89.3) | 25 (89.3) |  | 25 (89.3) |  | 25 (89.3) |  | 25 (89.3) |  |
| Missing | 0 | 0 |  | 0 |  | 0 |  | 0 |  |
| CS + LABAs |  |  | <0.0001 |  | 0.0011 |  | 0.0052 |  | 0.0052 |
| Total | 28 (100.0) | 26 (100.0) |  | 26 (100.0) |  | 26 (100.0) |  | 26 (100.0) |  |
| Yes | 22 (78.6) | 20 (76.9) |  | 15 (57.7) |  | 13 (50.0) |  | 13 (50.0) |  |
| No | 6 (21.4) | 6 (23.1) |  | 11 (42.3) |  | 13 (50.0) |  | 13 (50.0) |  |
| Missing | 0 | 2 |  | 2 |  | 2 |  | 2 |  |
| Nasal CS + SABAs + Antihistamines |  |  | n.d. |  | n.d. |  | n.d. |  | n.d. |
| Total | 28 (100.0) | 28 (100.0) |  | 28 (100.0) |  | 28 (100.0) |  | 28 (100.0) |  |
| Yes | 0 (0.0) | 0 (0.0) |  | 0 (0.0) |  | 0 (0.0) |  | 0 (0.0) |  |
| No | 28 (100.0) | 28 (100.0) |  | 28 (100.0) |  | 28 (100.0) |  | 28 (100.0) |  |
| Missing | 0 | 0 |  | 0 |  | 0 |  | 0 |  |
| ^1^Chi-square test; ^2^Final visit; ^3^If information from the last visit was missing, the same information from the 12-month visit was assigned to the same patient; ^4^Calculation is not possible due to missing values in a category.  Abbreviations: CS, corticosteroids; n.d., not determined; LABAs, long-acting B2 agonists; LOCF, last observation carried forward; SABAs, short-acting B2 agonists. | | | | | | | | | |
|  |  |  |  |  |  |  |  |  |  |
