# Supplementary material for: Effectiveness and safety of subcutaneous immunotherapy using a depigmented, polymerized extract of cat epithelium in allergic patients: a retrospective, real-world study
Source: Front Allergy. 2025 Sep 18;6:1642315. doi: 10.3389/falgy.2025.1642315 (PMC12488638; doi:10.3389/falgy.2025.1642315)
Supplement: Supplementary file 5 [file Table5.docx]

| **Supplementary Table S5. Immunological data** | | | | | | | | | | |  |  |  |  |  |  |  |  |
| --- | --- | --- | --- | --- | --- | --- | --- | --- | --- | --- | --- | --- | --- | --- | --- | --- | --- | --- |
|  | **Visit** | | | | | | | | | |  |  |  |  |  |  |  |  |
| **Variable** | **Baseline** | **6 months** | p-value^1^ | **12 months** | p-value | **18/24 months^2^** | p-value | **Final LOCF^3^** | p-value |  |  |  |  |  |  |  |  |  |
| Cat epithelium‑specific IgE^4^ |  |  | 0.1246 |  | 0.0314 |  | 0.7086 |  | 0.2305 |  |  |  |  |  |  |  |  |  |
| N (%) | 27 (100.0) | 10 (100.0) |  | 10 (100.0) |  | 11 (100.0) |  | 19 (100.0) |  |  |  |  |  |  |  |  |  |  |
| Mean (SD) | 47.17 (33.88)^5^ | 43.93 (30.93) |  | 23.67 (17.38) |  | 42.32 (39.08) |  | 35.12 (32.70) |  |  |  |  |  |  |  |  |  |  |
| Median (Q1; Q3) | 42.5 (10.5; 78.0) | 39.2 (25.1; 58.8) |  | 17.5 (9.1; 37.4) |  | 30.1 (9.5; 72.8) |  | 30.1 (9.1; 55.7) |  |  |  |  |  |  |  |  |  |  |
| Min-Max | 1.5-100.0 | 1.8-97.9 |  | 6.3-57.7 |  | 1.4-106.8 |  | 1.4-106.8 |  |  |  |  |  |  |  |  |  |  |
| Missing, n | 1 | 18 |  | 18 |  | 17 |  | 9 |  |  |  |  |  |  |  |  |  |  |
| Total IgE |  |  |  |  |  |  |  |  |  |  |  |  |  |  |  |  |  |  |
| N (%) | 27 (100.0) | 10 (100.0) |  | 10 (100.0) |  | 11 (100.0) |  | 19 (100.0) |  |  |  |  |  |  |  |  |  |  |
| Mean (SD) | 557.40 (710.84) | 473.35 (444.92) |  | 330.81 (413.28) |  | 328.90 (328.14) |  | 343.46 (377.67) |  |  |  |  |  |  |  |  |  |  |
| Median (Q1; Q3) | 294 (127.0; 726.0) | 271.3 (178.8; 682.1) | 0.7948 | 176.2 (125.0; 285.3) | 0.3339 | 216.9 (70.4; 466.5) | 0.6618 | 215.6 (79.1; 466.5) | 0.2884 |  |  |  |  |  |  |  |  |  |
| Min-Max | 24.5-3431.0 | 74.8-1275.5 |  | 50.0-1419.8 |  | 14.4-1006.6 |  | 14.4-1419.8 |  |  |  |  |  |  |  |  |  |  |
| Missing, n | 1 | 18 |  | 18 |  | 17 |  | 9 |  |  |  |  |  |  |  |  |  |  |
| Anti-Fel d 1 IgE |  |  | 0.8611 |  | 0.2904 |  | 0.7592 |  | 0.7101 |  |  |  |  |  |  |  |  |  |
| N (%) | 26 (100.0) | 10 (100.0) |  | 10 (100.0) |  | 11 (100.0) |  | 19 (100.0) |  |  |  |  |  |  |  |  |  |  |
| Mean (SD) | 27.46 (34.57) | 30.96 (27.87) |  | 12.70 (9.83) |  | 30.27 (30.91) |  | 22.87 (25.66) |  |  |  |  |  |  |  |  |  |  |
| Median (Q1; Q3) | 20.9 (3.5; 31.3) | 20.0 (16.2; 49.0) |  | 12.7 (4.7; 16.6) |  | 17.8 (5.9; 45.7) |  | 16.6 (4.7; 30.4) |  |  |  |  |  |  |  |  |  |  |
| Min-Max | 0.1‑143.6 | 1.8‑95.8 |  | 1.2­‑33.1 |  | 0.2‑97.1 |  | 0.2‑97.1 |  |  |  |  |  |  |  |  |  |  |
| Missing, n | 2 | 18 |  | 18 |  | 17 |  | 9 |  |  |  |  |  |  |  |  |  |  |
| Cat epithelium‑specific IgG4 |  |  |  |  |  |  |  |  |  |  |  |  |  |  |  |  |  |  |
| N (%) | 26 (100.0) | 10 (100.0) |  | 10 (100.0) |  | 11 (100.0) |  | 19 (100.0) |  |  |  |  |  |  |  |  |  |  |
| Mean (SD) | 3.84 (5.92) | 5.34 (5.61) |  | 5.88 (6.40) |  | 4.43 (4.89) |  | 5.49 (5.72) |  |  |  |  |  |  |  |  |  |  |
| Median (Q1; Q3) | 1.9 (0.5; 3.2) | 3.1 (1.1; 12.4) | 0.0128 | 3.4 (2.1; 9.8) | 0.5831 | 3.5 (0.9; 5.9) | 0.0092 | 3.7 (1.6; 9.8) | 0.0306 |  |  |  |  |  |  |  |  |  |
| Min-Max | 0.0-23.6 | 0.3-14.2 |  | 0.1-20.0 |  | 0.1-16.3 |  | 0.1-20.0 |  |  |  |  |  |  |  |  |  |  |
| Missing, n | 2 | 18 |  | 18 |  | 17 |  | 9 |  |  |  |  |  |  |  |  |  |  |
| Anti-Fel d 1 IgG4 |  |  | 0.0212 |  | 0.9196 |  | 0.1083 |  | 0.3549 |  |  |  |  |  |  |  |  |  |
| N (%) | 26 (100.0) | 9 (100.0) |  | 10 (100.0) |  | 11 (100.0) |  | 19 (100.0) |  |  |  |  |  |  |  |  |  |  |
| Mean (SD) | 3.12 (5.33) | 5.25 (6.87) |  | 4.34 (4.47) |  | 3.01 (3.74) |  | 3.91 (4.18) |  |  |  |  |  |  |  |  |  |  |
| Median (Q1; Q3) | 0.4 (0.2; 1.8) | 1.5 (0.4; 11.1) |  | 2.7 (0.5; 9.7) |  | 1.3 (0.7; 5.2) |  | 2.5 (0.7; 5.6) |  |  |  |  |  |  |  |  |  |  |
| Min-Max | 0.0-18.3 | 0.2-18.8 |  | 0.1-11.8 |  | 0.1-12.8 |  | 0.1-12.8 |  |  |  |  |  |  |  |  |  |  |
| Missing, n | 2 | 19 |  | 18 |  | 17 |  | 9 |  |  |  |  |  |  |  |  |  |  |
| ^1^Student t-test for paired data; ^2^Final visit; ^3^If information from the last visit was missing, the same information from the 12-month visit was assigned to the same patient; ^4^IgE levels expressed in kU/L; ^5^Subjects were considered sensitized if IgE >0.35 kU/L.  Abbreviations: IgE, immunoglobulin E; IgG, immunoglobulin G; LOCF, last observation carried forward; SD, standard deviation; Q1, first quartile; Q3, third quartile. | | | | | | | | | | |  |  |  |  |  |  |  |  |
